# Spatial variation in the mutation rate within the plant shoot apical meristem

**DOI:** 10.1073/pnas.2514507122

**Published:** 2025-11-10

**Authors:** Kirk R. Amundson, Mohan P. A. Marimuthu, Oanh Nguyen, Konsam Sarika, Isabelle J. DeMarco, Angelina Phan, Isabelle M. Henry, Luca Comai

**Affiliations:** ^a^Department of Plant Biology and Genome Center, University of California, Davis, CA 95616; ^b^Department of Biology, University of Massachusetts Amherst, Amherst, MA 01003; ^c^Indian Council of Agricultural Research, Research Complex for North Eastern Hill Region, Manipur Centre, Imphal, Manipur 795004, India

**Keywords:** mutation, variation, development, germline, regeneration

## Abstract

The potential impact of new mutations on organismal function and evolution depends on the developmental fate of the affected cells. Stem cells in the shoot apical meristem form much of the plant body, but only those in the L2 can form gametes. This raises the question of whether the mutation rate is optimized for this developmental plan. To test this, we compare stem cell mutations accumulated over decades of clonal propagation in two potato varieties with those arising during leaf-cell regeneration. By sequencing DNA from layer-enriched cell fractions, we found that variant allele frequencies in whole-leaf DNA can predict a mutation’s layer of origin. We further observed that mutations accumulate independently in L1 but jointly in L2 and L3. In cultivar Desiree, L1 mutation rates were fourfold higher than in deeper layers, suggesting germline-progenitor protection. In Red Polenta, layer-specific DNA from regenerants marked by a periclinal chromosomal translocation revealed a similar, though subtler, bias, with L1 exhibiting a 1.6-fold higher rate. In stark contrast, mutations during regeneration of differentiated cells were fiftyfold more frequent and bore a pronounced 8-oxoguanine signature confined to intergenic regions. Across all samples—and consistent with enhanced repair or purifying selection—the genic mutation rate was less than half that of intergenic regions. These findings indicate that plants employ mechanisms to restrict mutations according to cellular context and genomic region and suggest that the layered organization of angiosperm meristems evolved to balance genetic fidelity with adaptability

## Significance

The plant body is formed by small clusters of stem cells located at growth points, the meristems. New plant types can emerge only if mutations affect gametic and meristematic cells. Further, the shoot apical meristem consists of three cell layers, L1, L2, and L3, that form different plant parts. Mutations in the L2 are most impactful because the L2 forms the germline and much of the somatic tissue. We show that the position in the plant body greatly affects the probability and type of mutation. Specifically, the epidermal layer evolves more rapidly than the L2. We propose a model in which plant meristems’ layered architecture optimizes the balance between genetic fidelity and adaptability, with implications for biotechnology and evolution.

**Fig. 1.**
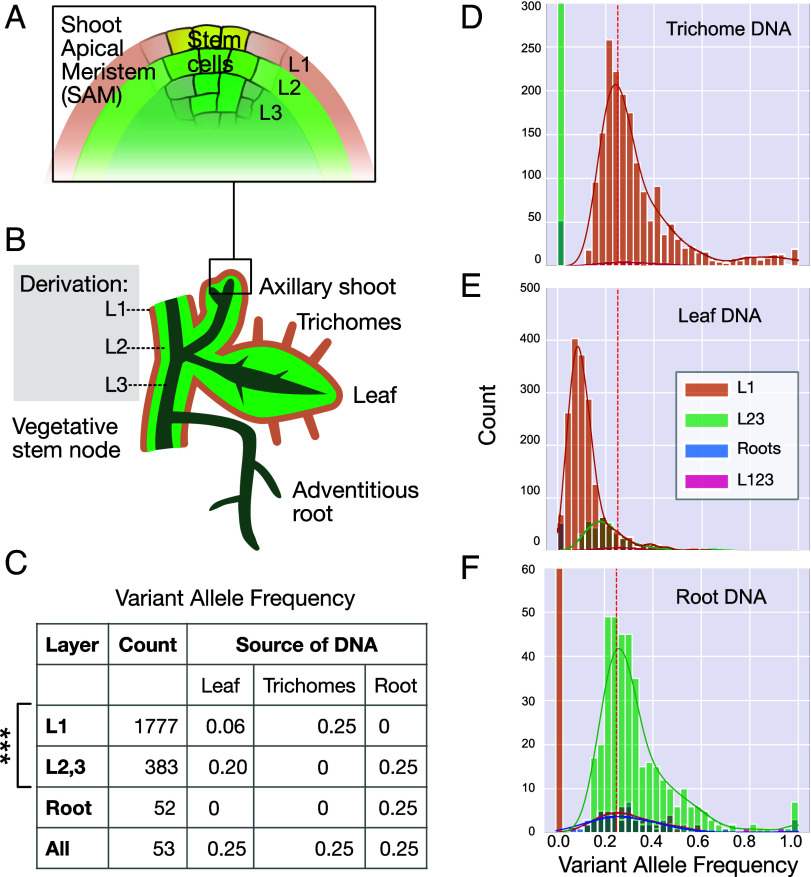
Detection of layer-specific mutations in tetraploid potato var. Desiree. (*A*) Plant SAM layered architecture. (*B*) Layer contribution to the plant body exemplified on the nodal unit of clonal propagation. While the seedling root is formed by the root meristem, the adventitious roots are formed from L3-derived tissue. (*C*) Count and frequency for mutations in different layer classes. The *Root* and *All* categories encompass mutations that likely escaped filtering, or, in the case of *Root* ones could be candidates for L3 mutations. *All* category mutations are ancestral. (*D*–*F*) The observed means for Single Nucleotide Variation (SNV) Variant Allele Frequency (VAF) reflect the proportion of cells in the sampled tissue that originate from each SAM layer and the corresponding dilution of the mutation. VAF of L1-derived SNVs is 25% in trichome DNA (*C* and *D*), but 6% in leaf DNA (*C* and *E*). Leaf and root share SNV (*C*, *E*, and *F*) at 20% and 25% VAF, respectively. The hatched line in *D*, *E*, and *F* represents the 25% VAF expectation for a simplex mutation in a tetraploid. Higher VAF, such as 1, represent haplotypes that have fewer than 4 copies due to SV or high haplotypic divergence.

The above-ground plant body derives from the proliferation of shoot apical meristems (SAM) that are organized by a small cluster of slowly dividing stem cells. In eudicots, the SAM is arrayed in three cell layers: L1, L2, and L3 (Fig. 1 *A* and *B*) (1–3). The L2 cells form the spores, which give rise to a segregated germline within the reduced haploid gametophyte. As a result, the L2 is the developmental precursor to the germline, while also forming much of the parenchyma of leaves, stems, and flowers. The L1, which is delineated early in embryogenesis, forms cells that mediate the interaction of the plant with the environment, including stomata, epidermis, and trichomes. In addition, cells derived from the L1 communicate with the underlying tissues, sharing metabolites and regulators and influencing plant development (4–6). The L3 forms ground tissue and the vascular system. The primary root of a seedling is formed by the root meristem. Adventitious roots, however, are the only roots formed during growth of a vegetatively propagated plant and emerge entirely from the L3 (7). L1, L2, and L3 are typically separated through L1 and L2 cell divisions occurring primarily in the anticlinal plane. However, periclinal cell divisions occur spontaneously or in response to injury, leading to displacement of one meristem layer by another (3, 8–10). Through layer displacement, meristematic cells in any layer can potentially give rise to the germline.

How does a mutation, which happens in a single cell, become somatically or sexually heritable? The persistent role of meristems in seed plant development has important implications on the transmission of mutations to the next generation. Somatic inheritance of a mutation requires that the affected stem cell or its descendants overtake a SAM. During this process, both mericlinal (wedge) and periclinal (layer) chimeric states are expected (1, 11, 12). Analysis of plant development in chimeras and after seed mutagenesis indicate the presence of two to four initial stem cells per layer (2, 3, 12). Mutation in one of these initials results in a radial sector (such as 1/3) affecting a subset of the nodes formed after the mutational event. The axillary meristems forming at the nodes can be uniformly mutant resulting in a nonsectored branch (ramet). Sexual inheritance is only likely for mutations that occur in the L2 initials. In contrast with organisms with an early segregated germline, such as mammals, plant mutations can overtake the soma in the same generation (13) and, if dominant or dosage sensitive, they can influence fitness.

Mutation rate is thought to depend on environment, cell division rate, DNA repair, and selection. Selection can act at the meristematic, somatic, and ramet level (14, 15). The mutation rate itself is an evolutionary trait set for each cell type to balance the advantage of variation with the need for genetic fidelity. Accordingly, mutations in terminally differentiated cells are thought to be more common than in germline cells (15, 16), and genes likely display reduced mutations compared to intergenic space (17, 18). While mutations in stem cells are rare and presumably shielded by wild-type alleles, clonal variants that exhibit new traits are relatively common and can often be horticulturally valuable (13).

There is little information on the spatial distribution of the mutation rate within the SAM stem cells, hampering our understanding of how mutations shape evolution at the clonal and sexual level. Here, we explore mutation accumulation in two potato clones using layer enrichment and isolation by single cell regeneration. Our results indicate that mutations partition into two compartments, derived, respectively, from the L1 and the L2,3, and accumulate at 1.6 to 4.5X higher rates in the L1-derived compartment. Compared to stem cells, differentiated cells display mutation signatures consistent with the action of reactive oxygen species. Genic DNA, however, appears protected in all tested cell types. This discovery furthers our understanding of how mutational mechanisms contribute to plant somatic and sexual evolution.

## Results

### Pure and Mixed Tissue Sources of Layer-Specific Mutations Reveal Epidermal (L1-derived) Bias.

To address the spatial pattern of mutations in potato clones, we analyzed mutations in tetraploid potato variety Desiree (*SI Appendix*, Fig. S1). After sequencing the leaf DNA, we mapped short reads to a primary assembly of tetraploid potato variety Red Polenta (described below) and identified Single Nucleotide Variants (SNV) and short indels that were absent in its parents, Urgenta and Depesche. We observed 383 variants with Variant Allele Frequency (VAF) around 0.2. In addition, we observed 4.6 times as many mutations (1,777) with a VAF of ~0.06. In an autotetraploid, a new mutation is expected in simplex dosage (1 of 4 alleles) and a corresponding VAF of 0.25. A lower than expected VAF could be caused by chimerism. Mutations could be unique to groups of cells in the sampled tissue and be eventually lost (12). However, independent sampling of the same clone indicated persistence of the mutations and consistency of the associated VAF (*SI Appendix*, Fig. S2). A possible explanation is that mutations accumulated in tissue derived from specific SAM layers and that the reduced VAF resulted from dilution of each layer’s DNA in the total preparation (Fig. 1 *A* and *B*). To test this hypothesis, we harvested trichomes from stem surfaces of Desiree to provide an enriched source of epidermal cell DNA and compared it to leaf DNA (derived from all three layers) and to root DNA, which is predominantly formed by the L3 (Fig. 1*B*).

In these three sample types, VAF values displayed distinct distributions (Fig. 1*C*), which enabled assignment to different layers. Because these mutations were fixed, we concluded that they originated in the SAM and refer to them by the layer of origin. L1 mutations were absent in roots, exhibited 0.06 VAF in leaves and 0.25 in trichomes. A second mutation class averaged 0.25 VAF in roots, 0.2 VAF in leaves, and were absent in trichomes (Fig. 1 *C*–*F*). We classified them at L2,3 hypothesizing that their presence in both leaf and root resulted from layer invasion events between L2 and L3 that created a homogeneous L2,3. The L1 and L2,3 classes were validated by their presence in two samples with frequencies consistent with layer anatomy. Interestingly, we found 4.6X more mutations in the L1 (1,777) than in the L2,3 (383), suggesting differential mutation accumulation between meristematic layers. Fifty-two mutations were unique to the root (Fig. 1*C*): Examining their mapping we concluded that 12 were contaminating reads. The rest could have originated recently in the L3. In addition, we detected 53 ancestral mutations that were present in all DNA samples at 0.25 VAF. The analysis of Desiree variation could be affected by unknown factors and bias (19). Accordingly, we searched for validation in another potato variety: tetraploid Red Polenta (*SI Appendix*, Fig. S1), (20, 21).

### Marking Layer Genomes Through Chimeric Polymorphisms.

**Fig. 2.**
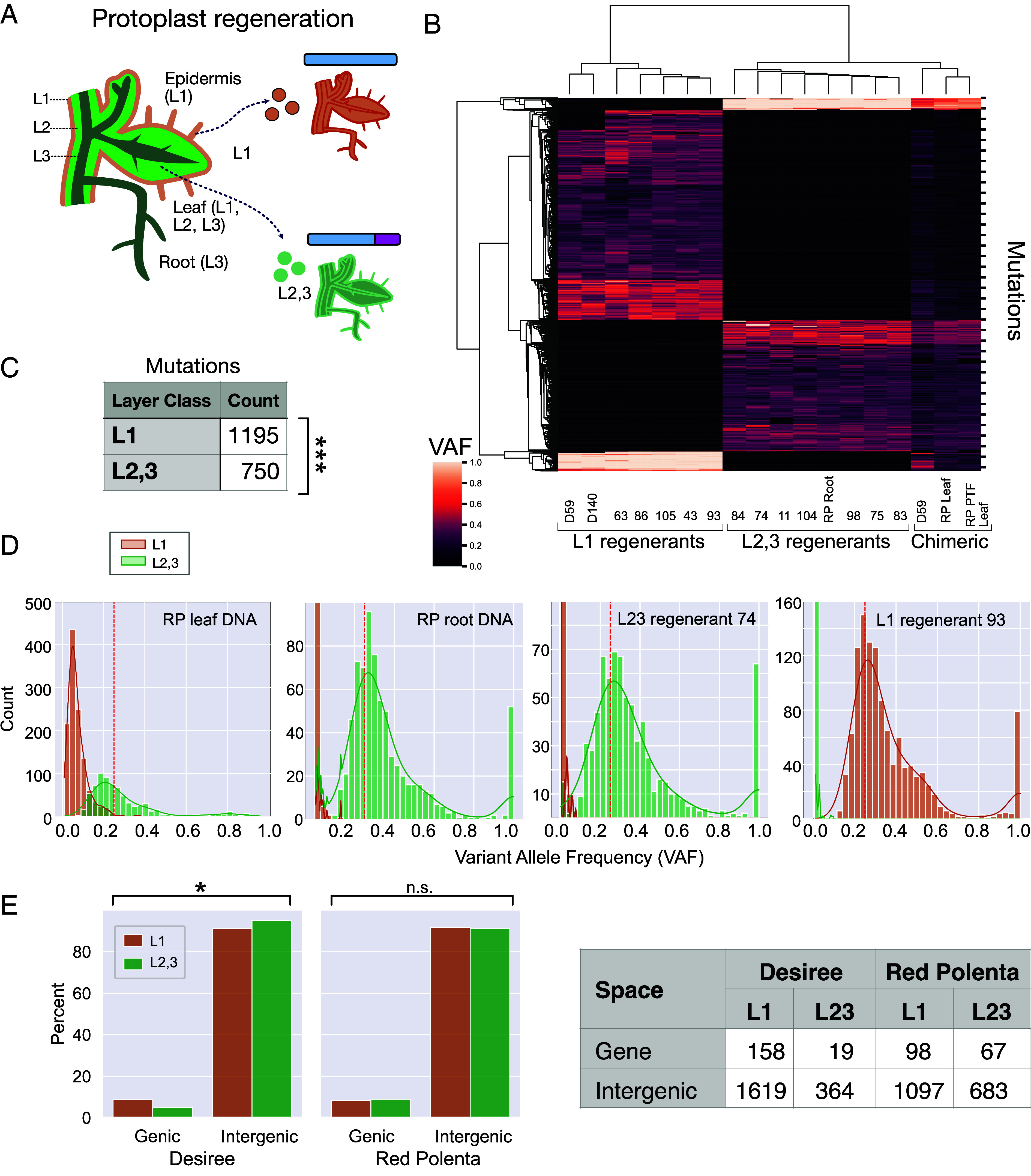
Protoplast regenerants of chimeric Red Polenta display layer-specific mutations. (*A*) L1-derived regenerants have normal chr.8 (blue), while those from L2,3 carry translocation tr8-7 (blue with red tip from chr.7). See also *SI Appendix*, Fig. S3. (*B*) Clustering of protoplast regenerants according to VAF reveals two distinct classes corresponding to the layer of origin. (*C*) Mutations detected in each layer of Red Polenta. (*D*) Distinct VAF profiles in DNA from different sources. In the original RP leaf DNA, mutations from both layers are present, but are diluted according to tissue representation. Root and protoplast regenerants exhibit single-layer origin with the expected 0.25 VAF. The hatched vertical line indicates the 0.25 VAF expected for a simplex mutation in a tetraploid. The smaller peak with high VAF is centered at 1.0 in roots (pure L3) and in regenerants (single cell origin, either from L1 or L2,3), but lower in leaf DNA of both Desiree and RP. The 1.0 VAF class of mutation is likely due to the mapping of sequencing reads to a single allele because the other haplotypes are likely deleted or too variant to map properly. In leaves and for this class of mutations, the cells derived from the L2,3 are mutant, while those derived from the L1 are not. The presence of L1-derived DNA in leaves accounts for the deviation from 1 because it provides the wild-type allele. (*E*) Comparison of mutation accumulation according to genomic space. For panel C, the statistical test is the χ2 test for goodness of fit with expected equal representation. For panel *E*, the χ2 test of independence was applied to the 2 × 2 table of each clone. The test highlights a statistically significant underrepresentation of mutations in the genic space of Desiree L2,3 compared to Desiree L1. *P < 0.05.

We found a similar layer-specific VAF distribution by sequencing leaf DNA of Red Polenta, a different variety that was separated from its clonal ancestor Urgenta over 60 y (*SI Appendix*, Fig. S1) (20). Studying variation in Red Polenta, we realized that it provided the opportunity for robust validation of layer-specific variation. Red Polenta carries a translocation (tr8-7) in which a terminal 4.6 Mb region of chr. 8 is replaced by the terminal 5.6 Mb of chr. 7 (Fig. 2*A*) (21). Tr8-7 was absent from one Urgenta clone, but present in two others that diverged ~25 y ago (20). We determined that tr8-7 is present in L2 and L3-derived cells but absent in cells derived from the L1 (*SI Appendix*, Fig. S3 *A* and *B*), conveniently providing a simple marker for layer identification. Its presence in the L2 was confirmed by segregation in the progeny (*SI Appendix*, Fig. S3*C*) and its presence in the L3 by cytological observation and bulk sequencing of shoot-borne roots (*SI Appendix*, Fig. S3*A*) (21). The L1, on the other hand, displayed four normal copies of chr.8, including a haplotype with 13,542 SNP (hap8-1), which is absent in cells carrying tr8-7, common among diverse potato germplasm, and inherited as a single haplotype in L1 protoplast-regenerated line MF93 (*SI Appendix*, Figs. S3*B*, S4, and S5). Hap8-1, but not tr8-7, was also present at 0.25 VAF in Urgenta, in Red Polenta trichome DNA (*SI Appendix*, Fig. S3*D*), and at 0.07 VAF in a second Red Polenta clone that had been independently propagated for ~25 y (*SI Appendix*, Fig. S6). On the other hand, if only Red Polenta leaf DNA sequence were to be available, this L1-specific simplex could easily be missed because mutations with low VAF (0.06) are often filtered.

### Single-Cell Regenerants Identify Unique Layers.

We leveraged the mutually exclusive hap8-1 and tr8-7 markers to identify the origin of single protoplast regenerants of Red Polenta (22). We found 6 regenerants with hap8-1 and 7 regenerants with tr8-7, indicating origin from L1 and L2,3, respectively (*SI Appendix*, Figs. S1 *A* and *B*, S3*E*, and S7). To assess the effect of the reference genome, we used Red Polenta long reads from PacBio and standard tools (*SI Appendix*, *Methods*) to produce a high continuity assembly (N50=45.4 Mb, 98.3% completeness of Solanales BUSCO genes, *SI Appendix*, *Methods* and Table S3 and Fig. S8). To call mutations, we compared layer-specific (i.e., shared by regenerants) alleles to the ancestral allele in two clones of cv. Urgenta without tr8-7. Using these criteria and stringent quality filtering (*SI Appendix*, *Methods* and Fig. S2), we identified 1,945 layer-specific mutations (Fig. 2*B*). These mutations displayed similar VAF distributions as the layer-specific mutations in Desiree, with additional information provided by the progeny clones: L2 and L3 mutations were indistinguishable, presumably because of rapid L2-L3 homogenization. Consistent with our analysis of Desiree, layer-specific mutations were significantly L1-biased, with 1.64-fold more detected mutations specific to L1 (1,195 for the L1 vs 750 for the L2,3) (Fig. 2 *C* and *D*). We further confirmed the location of these mutations by progeny analysis: Red Polenta L1 mutations were not transmitted while L2,3 mutations appeared in the progeny with VAF mean at 0.25 (*SI Appendix*, Fig. S9). We concluded that more mutations accumulated in the L1 than in the L2,3.

### Underrepresentation of Mutations in Genes.

To explore what sequence context was most often affected by mutations, we compared mutation rates in genic and intergenic regions based on SNPEff classification (23). Mutation rates were normalized by the sizes of the genomic regions under consideration (*SI Appendix*, *Methods*) and by the approximate divergence times between clones. We recognize the caveats of estimating somatic mutation rates per unit time. This estimate was used to provide a way to compare samples by deriving a per-site, time-based mutation rate. Comparison of layers by genomic space was significant for Desiree (Fig. 2*E*), with the L2,3 displaying underrepresentation of genic mutations, but overrepresentation of intergenic mutations. Red Polenta did not display a significant bias. Interestingly, regardless of layer, the mutation rate (mut * bp^−1^ * y^−1^) in L1 was 1.5 to 8X that of the L2,3 (Table 1). The mutation rate for intergenic DNA was 2 to 4X that of genic regions (Table 2). In summary, comparing the layers, Desiree exhibited strongly decreased accumulation in L2,3 compared to Red Polenta. Comparing genomic space, both varieties and layers displayed strong underrepresentation of genic mutations compared to intergenic DNA (Tables 1 and 2).

**Table 1.** Mutation rate comparison

Strain	Space	Bp	Years	Count			Mutation * bp^−1^ * y^−1^			L1/L2,3
				L1	L2,3	Regen	L1	L2,3	Regen	
Desiree	Gene	133000000	34	158	19		3.49E−08	4.20E−09		8.32
	Intergenic	547467000	34	1,619	364		8.70E−08	1.96E−08		4.45
Red Polenta	Gene	144046000	60	98	67		1.13E−08	7.75E−09		1.46
	Intergenic	657050000	60	1,097	683		2.78E−08	1.73E−08		1.61
										**Regen/L23**
Red Polenta regenerant 105	Gene	144046000	1			64			4.44E−07	57.31
	Intergenic	657050000	1			920			1.40E−06	80.82
Red Polenta regenerant 83	Gene	144046000	1			70			4.86E−07	62.69
	Intergenic	657050000	1			674			1.03E−06	59.21
Red Polenta regenerant 63	Gene	144046000	1			3			2.08E−08	1.20
	Intergenic	657050000	1			33			5.02E−08	2.90

**Table 2.** Genic mutation rate bias according to tissue type

Ratio	Des* L1	Des L2,3	RP L1	RP L2,3	Reg 105	Reg 83	Reg 63
Intergenic/gene	2.49	4.65	2.45	2.23	3.15	2.11	2.41

Des: Desiree; RP: Red Polenta; Reg: regenerant.

### Mutation Spectrum Differs Between Stem Cells and Single-Cell Regenerants.

**Fig. 3.**
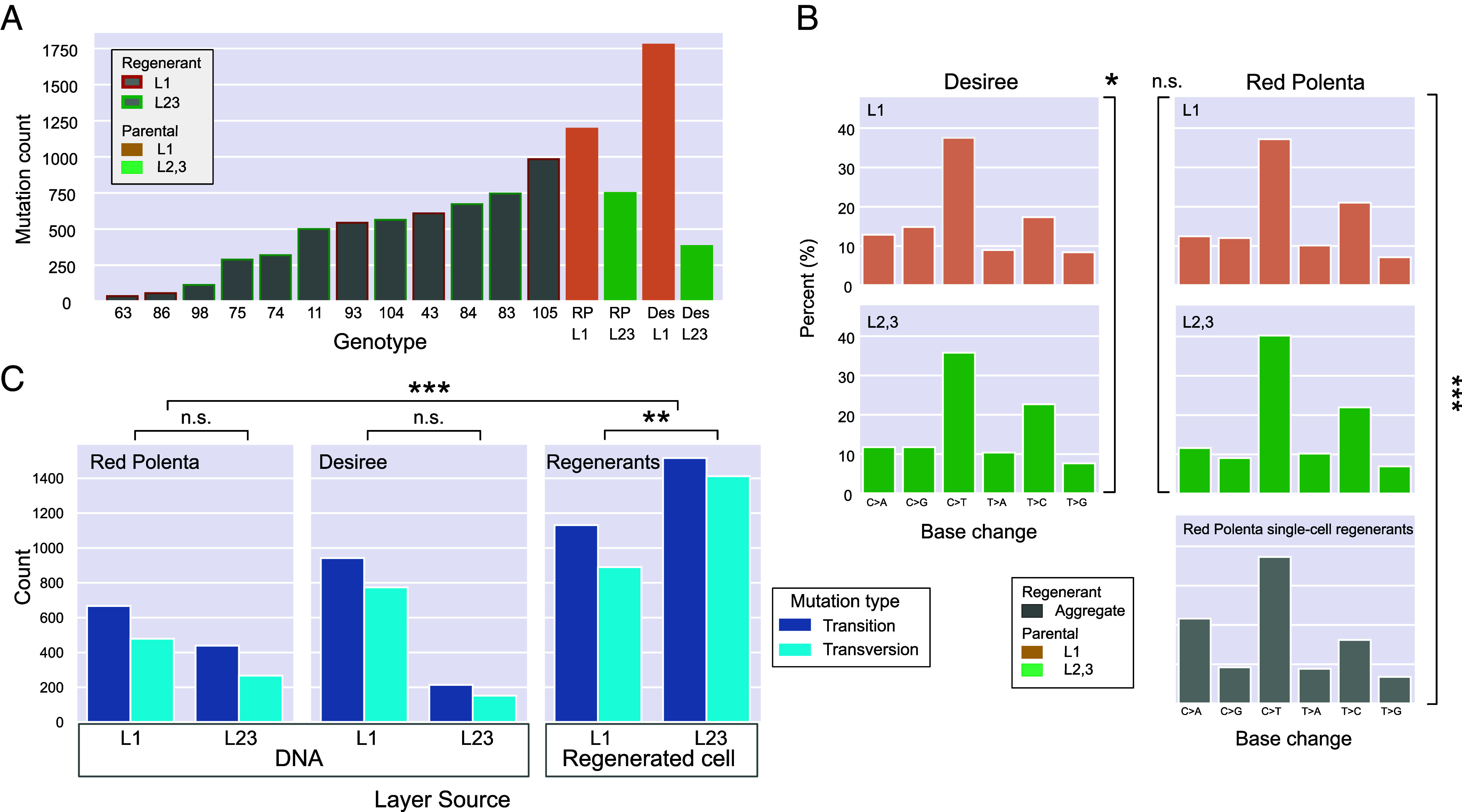
Mutation type and count in parental clones and Red Polenta protoplast regenerants. (*A*) Mutations accumulated in Desiree (D) and Red Polenta (RP) in, respectively, 34 and 60 y of vegetative propagation versus mutations accumulated during regeneration of protoplasts. (*B*) Base change profiles by clone. Compared to parental clone mutations, regenerants display a large bias toward C > A base change. (*C*) Transition and transversion mutations differ significantly according to layer origin of the regenerants and between private mutations of the regenerants and fixed mutations of varieties Desiree and Red Polenta. The χ2 test of independence tests the hypothesis that the observed differences are due to random chance and therefore rows and columns are not associated. See Table 2 for details. **P < 0.01; ***P < 0.001.

The biased accumulation in the L1 could result from differential exposure to environmental mutagens. To test this hypothesis, we compared the single-nucleotide spectrum in relation to layers. The frequencies of the six possible single nucleotide changes were significantly different in Desiree for L1 and L2,3, but not in RP, but the trend did not appear to be biologically relevant (Fig. 3 *A* and *B*, compare RP and Desiree). For both Desiree and Red Polenta, transition and transversion frequencies were not different according to layer (Fig. 3*C* and *SI Appendix*, Fig. S10). Trinucleotide spectra were consistent with these observations (*SI Appendix*, Fig. S11). In conclusion, the single-nucleotide mutation profile did not support the possibility of higher exposure to mutagens in the L1.

**Fig. 4.**
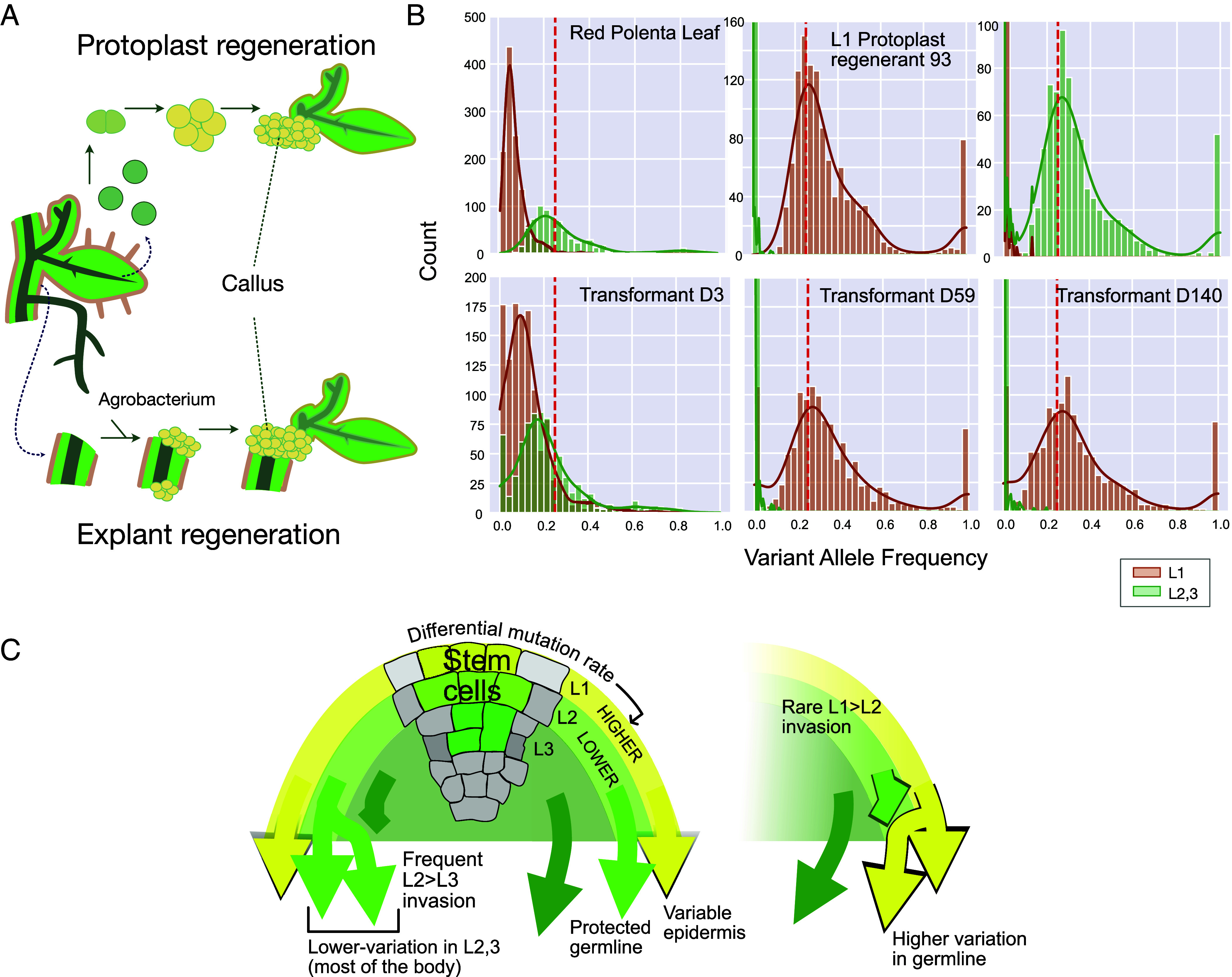
Layer of origin of Red Polenta explant regenerants and model of SAM function. (*A*) Regeneration methods: Leaf protoplasts are single cells resulting from cell wall digestion. Although differentiated, they can be induced to proliferate, form a callus, and regenerate. Explant regeneration is the common method for Agrobacterium-mediated transformation. In potato, it involves internodes, which are cultured on callus-inducing medium. (*B*) The three samples in the *Top* row serve as controls demonstrating the expected patterns for a chimeric (RP leaf), and nonchimeric (Regenerant 93 and RP root). The *Bottom* row displays three transformants of Red Polenta, which were produced by stem explant cocultivation and regeneration. Their pattern classifies them as, respectively, chimeric, and from L1 origin (last two; see also Fig. 2*B*). (*C*) Model of SAM function. During normal SAM growth, mutations accumulate differentially. Our data do not distinguish the origin of the L2,3. We postulated that L2 is most efficient in preventing mutation accumulation. Frequent invasion by the L2 of the L3 would ensure low variability in much of the soma while restricting costly fidelity-maintaining mechanisms to the L2. The probability of L2 invasion by the L1 is much lower, but significant enough to ensure that L1 innovations with high fitness could enter the sexual cycle.

Our regenerated material enabled comparison between two different sets of mutations: those formed in stem cells during normal vegetative growth, which were shared within a layer-specific regenerant set (Fig. 2*A*), and those formed immediately before or during protoplast regeneration (Figs. 3 and 4*A*), which were private to each regenerant. In 12 regenerants (5 for L1, 7 for L2,3), we measured widely different mutation numbers per individual, ranging between 36 and 984. In all but one regenerant, the VAF peaked at the expected 0.25 VAF (*SI Appendix*, Figs. S12–14) consistent with tissue homogeneity and lack of genetic separation in the layers. Protoplast regeneration involves the induction of totipotency upon single differentiated leaf cells. Accordingly, the detected mutations could have accumulated during leaf cell differentiation, protoplasting, or early regeneration.

Equating the yearly clonal cycle of vegetative growth to one generation, we estimated the genic mutation rate in the L2,3 at 4.2E−9 (base^−1^ * generation^−1^) for Desiree and 5.9E−9 for Red Polenta. It was 1.5 to 8X higher in the L1 (Table 1). In the regenerants, assuming that regeneration accounted for one generation, the mutation rate was as high as 50X that of the L2,3 (Table 1). In all but one case (the exception being clone 86, one of the regenerants with the lowest count of mutations), the mutation profile differed significantly from that of stem cell mutations by a large increase in GC>TA transversions (Fig. 3 *B* and *C*), consistent with formation of 8-Oxoguanine by the reaction of reactive oxygen species (ROS) with guanine.

### Variation in Layer Origin of Plants Regenerated From Explants.

Red Polenta protoplasts from both L1 and L2,3 cells regenerated efficiently (Fig. 2*A*). To assess which layer contributes to regeneration during stem explant cocultivation (Fig. 4*A*), one of the steps in transformation (24), we sequenced three Red Polenta (Fig. 4*B*) and thirty-four Desiree transformants and used the assay demonstrated in Fig. 2 to determine their origin. Two of the Red Polenta (D59 and D140) were from the L1, while the third (D3) maintained the chimeric pattern of the vegetative parent and thus originated from at least two cells that encompassed both layers (Figs. 2*B* and 4*B*). In contrast, all Desiree regenerants displayed the L2,3 mutations and lacked the L1 mutations, indicating origin from the L2,3 (*SI Appendix*, Fig. S15). We concluded i) that explant regeneration can potentially arise from all layers depending on genotype and treatment, ii) that regenerants can maintain chimericity, a potentially desirable feature in clonal species, and iii) that the two varieties strongly differed in the ability to regenerate from the L1: Desiree had effectively lost totipotency in the L1. Surveying our layer-specific mutations, only eight mutations in Desiree L1 were located in genes and predicted to have a high impact on function (*SI Appendix*, Table S8). One candidate mutation was a frameshift in a homolog of *ATHB-8*, which encodes an HD-ZIP III protein known to play a key role in *Arabidopsis* meristem regeneration (25).

## Discussion

The process of vegetative propagation entails formation of a new plant from a single stem node: The shoot is derived from an axillary meristem while roots emanate from the stem vascular cambium (also a meristem; Fig. 1 *A* and *B*). Under these conditions, mutations can only become established (fixed in the soma and clonally heritable) if they originate in stem cells. The study of these mutations leads us to the following conclusions: First, we demonstrated an effective method to explore spatial mutation patterns by sampling root, trichome, and leaf. Second, individual mutations accumulated either in the L1-derived cells or the L2,3-derived cells, but the same mutation did not occur in both. Third, mutations accumulated at higher frequency in the L1-derived cells compared to the L2,3-derived cells (4X in Desiree, 1.6X in Red Polenta). Fourth, the number accumulated in the L2,3-derived cells (383 for Desiree, 750 for Red Polenta) is proportional to the years of independent propagation separating the compared genomes (~34 for Desiree, ~60 for Red Polenta), yielding an L2,3 genic mutation rate of ~5.0E−9 (/bp/generation, Table 1). Fifth, by contrast, the L1 varietal rates differed (1,777 vs 1,195), indicating that variation is potentially common and that Desiree L1 accumulates mutations at a faster rate. Sixth, mutations are ¼ to ⅛ less frequent in genic DNA compared to intergenic (Table 2). This is a previously observed property (26), potentially attributable to negative selection (27) and differential protection (17, 18). Seventh, regeneration results predominantly from a single layer capturing the connected unique genetic context. Last, immediately before or during the relatively short time of regeneration from protoplasts, up to 887 mutations accumulated in the regenerating cell affecting uniformly all layers and displayed a different mutational fingerprint consistent with the action of ROS. Surprisingly, this fingerprint was absent in genic DNA of the regenerants and in the stem cell–derived mutations consistent with biased protection of genes in differentiated cells and in protection of the stem cell niche from oxidants.

Our analysis leveraged layer enrichment as well as clonal extraction through protoplast regeneration. In Desiree, fifty-three mutations were shared between layers, but could have resulted from mutations in subclones of either parent that were not sampled. In both varieties, the large majority of SNV partitioned into L1 vs L2,3. For Red Polenta, ad hoc genome assembly and replicated regenerants provide robust assessment of layer-differential mutations, confirming the observations based on comparison of trichomes vs root DNA.

The genetic behavior of the layers is consistent with the tunica-corpus proposal (28), but suggests that the tunica should consist only of the L1, and the corpus of the L2 and L3 as indicated for grapevine (29). Homogenization between L2 and L3 must take place in the SAM, but we have no direct evidence of its direction. Therefore, we cannot determine whether the lower mutation rate in the L2,3 corresponds to that of the L2 or L3. Literature on chimeric plants (10, 29, 30), on layer-specific regulation of genome protective pathways (31–33), and evolutionary considerations (15) suggest that L2, the germline precursor, is likely to have the lowest mutation rate. Given the frequent invasion of L3 by L2 reported for potato axillary meristems (10, 34), the L2,3 compartment likely displays the genetic load of the L2. Adventitious roots originate from L3 and thus their mutations are consistent with those of the L2,3. Depending on the frequency of L2-L3 invasion, it should be possible and important to measure the respective mutation rates.

In both tested varieties, we observed layer bias. Layer bias was reported in a recent report in apricot (35). Similar differential stability between layers has been described for transposon excision in peach (30). This layer bias could be caused by one or more of several mechanisms. i) Enhanced action of environmental mutagens such as UV radiation on the L1 due to increased exposure of the L1. The differential action of an environmental mutagen, however, was not detected by comparing the mutation spectra. ii) The L1 could divide at a faster rate than the L2. A developmental analysis of cell division rates is lacking for potato. In arabidopsis, the cell division rates of the L1 and L2 appear to be comparable (36, 37). Temperature, however, influences the number of cells in the L2-derived tissue, but not in the L1-derived tissue (38). It is possible, therefore, that the cell division rate could vary between the two layers depending on the growth environment. An interesting question is whether germline cells, potentially through segregated quiescence, could achieve an even lower rate than the clonal L2 (39). iii) Negative selection could be less severe in the L1. We could not test this hypothesis because the relatively small number of mutations in coding space hindered dN/dS comparative analysis. We noted a deficit of deleterious mutations in Desiree L23, but not in Red Polenta L23 (*SI Appendix*, Table S2 *J* and *K*). iv) The L2 could express genomic protection pathways at a higher level than the L1. Results in arabidopsis and maize are consistent with this possibility as genes involved in DNA repair and DNA methylation have been found to be expressed at higher levels in the L2 (31–33). In summary, differential regulation of genome stability and, potentially, cell division rates and negative selection should be considered high-priority candidate mechanisms. Our previous analysis of protoplast regenerants shows that loss of stem cell status in Red Polenta, i.e., cellular differentiation and tissue culture-mediated regeneration, is frequently associated with loss of genome stability (22). Here, we show that regenerants display a distinct mutation profile which, compared to the historically accumulated mutations, is enriched in CG>AT transversions and indicates oxidative DNA damage. Surprisingly, although the somatic mutation number was high in all regenerants, the number of mutations varied from regenerant to regenerant. Because all cells were exposed to the same regeneration treatment, this suggests that preexisting conditions, such as cell type, determine the severity of genomic instability.

What are the implications of increased mutation accumulation in the L1 and increased fidelity in the L2,3? Anecdotally, the L2,3-specific regeneration profile of Desiree explants and its high L1-specific mutational load, suggest a possible consequence of enhanced mutation accumulation in the L1: deterioration of the L1 genome leading to chimeric loss of totipotency. In Desiree, among the 8 genes affected by predicted severe mutations in the L1, Soltu.DM.08G014820.1 encodes a homolog of AtHB-8 and is a good candidate for a regeneration factor (25, 40): A dosage effect or perhaps expression of a truncated protein may decrease its function and compromise regeneration of the L1.

At a more general level, the assumption that the L2 displays maximum fidelity compared to the L1 and L3, suggests a model (Fig. 4*C*) in which the SAM minimizes metabolic cost of higher repair while extending reduced variability to both the germline (L2) and to the bulk of the soma through L2 invasion of L3. Chimeric variants including those in the surface cell layer could be subject to selection. Mutations accumulating during clonal growth are heterozygous and, typically, are thought to be neutral because of dominance of the wild-type allele. The probability of genetic shielding should be even higher in a tetraploid. Nevertheless, fitness effects due to dosage and heterozygosity are possible (41, 42). Two additional arguments support the potential for premeiotic effects of mutations: First, fitness effects by heterozygous mutations have been documented in humans (43, 44), yeast, and plants (42, 45), and, second, clonal variants are widespread in horticulture (13). A dominant or additive L1 mutation may change epidermal properties, providing tolerance to a pest or to an environmental stress (*SI Appendix*, Fig. S16), or by affecting development of other layers (5, 6, 46–49). If the L1 invades the L2 in the SAM (3, 29, 50–52), the connected mutations may enter into the sexual cycle (*SI Appendix*, Fig. S16).

In conclusion, our investigation reveals that through differential mutation accumulation within the meristematic layers, the plant SAM adopts a conservative strategy for the L2 and L3, while increased mutations in the L1 could result in higher variability in surface cells. Increased understanding of spatial dynamics of the mutational process will facilitate better understanding of how mutations contribute to both clonal and sexual evolution.

## Materials and Methods

We extracted genomic DNA samples from trichomes and shoot-borne root samples of greenhouse-grown Desiree clone Des-1, obtained from Joyce van Eck (Cornell University), and from Red Polenta (20). Leaf genomic DNA samples were generated for thirteen clones regenerated from Red Polenta leaf protoplasts (22) and obtained for Urgenta and Depesche from the IPK Potato Genebank (*SI Appendix*, Fig. S1). These samples were subjected to deep whole-genome short-read sequencing (*SI Appendix*, Table S1). Leaf gDNA samples for an S1 population of protoplast regenerant MF93 were sequenced at low coverage (*SI Appendix*, Table S1). Discovery and genotyping of tr8-7 and hap8-1 were carried out via read alignments to the DM1-3 v6.1 genome and previously described analyses (20, 21, 53, 54). For the Red Polenta genome assembly, we used 35× per haplotype coverage PacBio HiFi sequencing to construct a primary assembly with hifiasm (55). We then used the Extensive De-Novo TE Annotator (56) and RepeatMasker to soft-mask repeats in the primary assembly. To annotate genes, we used BRAKER3 (57) with RNA-seq of leaves from Red Polenta and MF93 with publicly available RNA-seq (58) and protein data (53, 59, 60) (see additional information in *SI Appendix*, *Methods*).

Periclinal mutations in Desiree were identified by read alignment to the primary assembly, followed by variant calling and filtering for mutations that were absent from one layer-enriched sample, present at any VAF in mosaic leaf tissue, and present with ≥8× read coverage and 0.125 VAF in the other layer-enriched sample. To identify periclinal mutations in Red Polenta, we required that a putative mutation was not detected in either USDA or IPK Urgenta clones, present with ≥8× coverage and ≥0.125 read support in at least four regenerants from one layer (either L1 or L2,3), and not detected despite ≥8× coverage in all regenerants from the other layer. To identify regenerant-specific mutations, we required that a mutation was present with ≥8× coverage in only one protoplast-regenerated line (19). All categories of mutations were subject to contaminant removal via BLASTN of all reads overlapping a putative mutation call to the NCBI nt database. If four or more reads overlapping a putative mutation aligned to a species other than *Solanum*, it was removed.

To calculate mutation rates, we tallied positions covered by 8 or more reads exceeding minimum mapping quality (20) and base quality (20) thresholds in the Red Polenta assembly for each sample. For Desiree, genomic positions were considered assayed if the minimum coverage thresholds were met for each of USDA Urgenta leaves, IPK Depesche leaves, and Desiree-1 trichomes, leaves, and roots. For Red Polenta, positions were considered covered if these criteria were met for Red Polenta, USDA Urgenta, and at least four regenerants from each layer. As a final filtering step, we removed sites with mean mapping quality ≤ 40 across all considered samples.

To determine the layers from which Red Polenta stem explants were regenerated, we determined the dosage of tr8-7 and hap8-1 from deep short-read sequencing of each line. For regenerated Desiree explants, the low-coverage sequencing generated by (22) was reanalyzed using the haplotype-based dosage framework adapted from ref. 54. Reads overlapping L1- or L23-specific mutations were aggregated across all loci, and the fraction of reads supporting layer-specific mutations were reported. Additional details on materials and methods can be found in *SI Appendix*.

## Supplementary Material

Appendix 01 (PDF)

## Data Availability

Raw sequencing reads are available on NCBI Sequence Read Archive under project PRJNA1241272 (61). The custom scripts used for analysis are available at GitHub (62, 63).
