# Supplementary material for: Spatial variation in the mutation rate within the plant shoot apical meristem
Source: Proc Natl Acad Sci U S A. 2025 Nov 10;122(46):e2514507122. doi: 10.1073/pnas.2514507122 (PMC12646271; doi:10.1073/pnas.2514507122)
Supplement: Supplementary file 1 — Appendix 01 (PDF) [file pnas.2514507122.sapp.pdf]

PNAS

Supporting Information for
Spatial variation in mutation rate within the plant shoot apical meristem

Kirk R Amundson[1,2], Mohan Prem Anand Marimuthu[1], Oanh Nguyen[1], Konsam Sarika[1,3], Isabelle J DeMarco[1], Angelina Phan[1], Isabelle M Henry[1], and Luca Comai[1]

Luca Comai
Email: lcomai@ucdavis.edu

This PDF file includes:

Supporting text
Figures S1 to S18
Tables S3, S9
SI References

Other supporting materials for this manuscript include the following:

Tables S1, S2, S4, S5, S6, S7, S8

Supporting Information Text

Plant materials and sequencing

Solanum tuberosum group Tuberosum variety Desiree-1 was obtained from Joyce Van Eck (Cornell University) as a single axenic cutting. Variety Red Polenta is a clonal descendant of variety Urgenta (1) obtained as a single axenic cutting (PI310467) from the US Potato Genebank. A previous report describing regeneration of the variety Desiree from protoplasts (2) used Red Polenta because of a variety swap at the Genebank presumably at the time of import, which was 1966 (1).

Cuttings were propagated *in vitro* under 16h light 25˚C: 8h dark 18˚C on half-strength Murashige and Skoog medium adjusted to pH 5.7 with KOH and supplemented with 1% sucrose, 1x Gamborg vitamins and 0.5g/L MES. Single-node cuttings were transferred to fresh media every 1-2 months. Red Polenta and protoplast-regenerated plants were continuously propagated by tubers on soil in 8" pots in ambient greenhouse conditions at Davis, CA, USA. Leaflets of the three youngest leaves from five tuber-propagated plants of each genotype (Red Polenta, MF93, MF43, MF83 and MF75) were collected for high molecular weight DNA isolation and library preparation. Seeds produced from self-pollination of MF93 were soaked in 2000 ppm GA3 for 24h, rinsed thoroughly with sterile water, surface sterilized in 50% commercial bleach for 5 minutes, and rinsed an additional five times with sterile water. Seeds were then germinated on half-strength Murashige and Skoog medium in the aforementioned growth chamber conditions. Cuttings were propagated every 1-2 months as described above.

High molecular weight DNA was isolated from 1.5 grams of leaves using a sorbitol pre-wash and high-salt CTAB extraction as previously described (3). Quality was assessed on a Bioanalyzer, and HiFi libraries was then prepared according to the manual "Procedure & Checklist - preparing HiFi SMRTbell Libraries using SMRTbell Express Template Prep Kit 2.0", including initial DNA fragmentation by g-Tubes (Covaris) and final library size binning by SageELF (Sage Science, Beverly, MA). Size distribution was controlled using a Bioanalyzer again before sequencing. Size selected libraries were sequenced on a Sequel IIe instrument at the University of California Davis DNA Technologies Core (Davis, CA, USA) with Binding kit 2.0 and Sequel II Sequencing Kit 2.0. For short read sequencing, genomic DNA was extracted from greenhouse or *in vitro* grown plants and used for library preparation as previously described (4). Roots were collected from washed root systems after careful separation of true adventitious roots from rhizomes. We extracted total RNA from leaves of Red Polenta and MF93 using a standard TRIzol protocol and then prepared cDNA libraries using the KAPA RNA Hyper Prep Kit (cat. no KK8581, Roche) according to manufacturer recommendations. Additional details on library preparation and sequencing are provided in Table S1. Sequencing reads from previous studies (1, 2, 5) were retrieved from the National Center for Biotechnology Information (NCBI) Sequence Read Archive (SRA) and incorporated in subsequent analyses.

Sequence processing and alignment to DM1-3 reference genome

Short reads were trimmed with Cutadapt 1.15 (6) and aligned to the DM1-3 version 6.1 assembly with BWA mem (version 0.7.12-r1039) (7). PCR duplicates were removed with Picard (version 2.18) MarkDuplicates. For all paired-end read alignments with overlapping mates, the overlap region was soft-clipped for one of the two mates using bamutil:clipOverlap (version 1.0.14) (8). Paired ends with mates aligning to different chromosomes were removed using a custom awk script. Alignments processed in this way were used for read depth analyses and all variant calling based on alignments to DM1-3.

Layer-specific allele discovery and genotyping

Variant discovery

Copy number was inferred from median read depth of non-overlapping 10kb windows of the DM1-3 reference genome (9) as previously described (1). Upon observing polymorphism with respect to tr8-7, we assayed all regenerants for single-nucleotide and short indel mutations. Variants were called and genotyped using freebayes (version 1.3.4) (10) with minimum mapping

quality 40, minimum base quality 20, population priors off, a maximum of six alleles considered per variant and all other parameters left at the default setting. To remove low-quality variants, the following hard filters were applied using bcftools (version 1.15) (11): TYPE='snp', NUMALT=1, EPP≤30, EPPR≤30, MQM≥30, MQMR≥30, RPP≤30, RPPR≤30, SAP≤30, SRP≤30. Only sites with Red Polenta read depth between 40 and 140 were retained. Novel variants associated with the presence of tr8-7 were defined as ≥12.5% VAF in each of the six regenerated lines that lacked tr8-7 and ≤12.5% VAF in each of the six regenerated lines that exhibited tr8-7. The 13,542 such SNPs located to the right of chr08:54,585,000 were defined as hap8.

Diversity panel variant genotyping
Publicly available short read sequencing of 102 partially redundant potato samples from previous studies (1, 4, 5, 12) was retrieved from NCBI SRA for read processing and alignment as described above. Processed alignments were used to genotype each of the 102 samples at the 13,542 hap8 SNP loci with freebayes version 1.3.4 by providing BED3 coordinates of each locus as targets with the --targets, and, to define alleles at each target, a VCF file containing only the target loci. We applied filters to retain only biallelic SNPs (NUMALT=1 & TYPE='snp') with ≤10% missing data to the resulting VCF, which retained variant calls at 5,980 loci. All samples corresponding to Red Polenta were removed, and all remaining duplicate samples were deduplicated, yielding a nonredundant panel of 76 samples. Allele frequencies were recalculated to represent the non-redundant samples (Red Polenta excluded) before plotting the allele frequency spectrum (Fig. S2).

Offspring genotyping
To assay tr8-7 dosage of Red Polenta S1 offspring, chromosome dosage analyses were carried out as previously described (2, 13). Selfed offspring exhibiting whole-chromosome aneuploidy for either chromosome 7 or 8 were excluded. For each remaining offspring (n=82), read depth in non-overlapping 1Mb windows of the reference genome was normalized to Red Polenta and then multiplied by four to account for tetraploidy. To define dosage genotypes, we averaged standardized coverage values of the dosage-variable 5.6 Mb and 4.6 Mb termini of chromosomes 7 and 8. For chromosome 7, dosage genotypes were defined as "down7" if the average standardized coverage was less than 3.9, "mid7" if between 3.9 and 4.75, and "up7" if greater than 4.75. For chromosome 8, dosage genotypes were defined as "down8" if average standardized coverage was less than 3, "mid8" if between 3 and 4.5, and "up8" if greater than 4.5. Among S1 progeny, we observed only "down7-up8", "mid7-mid8" and "up7-down8" haplotypes, which respectively correspond to 0, 1 or 2 copies of tr8-7.

To analyze hap8 dosage in MF93 S1 offspring, variants were jointly called using freebayes (version 1.3.4) with a BED file of 13,542 hap8 SNP coordinates provided as targets and a filtered VCF to define reference and alternate alleles. For each S1 offspring, read depth and hap8 allele-specific read depth were aggregated across all loci, and the corresponding proportion of hap8 specific alleles was used to determine dosage in each offspring: dosage 0 <5%, dosage 1 5-40%, dosage 2 40-60% and dosage 3 ≥60%.

Trichome DNA extraction and genotyping
Trichomes were isolated from five leaves harvested from a single greenhouse-grown plant. Leaflets and stipules were manually separated. Petioles were cut into 5 cm pieces, snap-frozen by immersion in a liquid nitrogen-chilled mortar and gently scraped with pre-chilled steel jewelers' forceps to remove trichomes. Leaflets were then flash frozen in the same mortar, and longer trichomes were gently scraped from the midrib, veins and adaxial surface with steel forceps. Visible leaf fragments or other debris were removed from the mortar. Trichomes from all five leaves were ground to a fine powder under liquid nitrogen for approximately 15 minutes and processed for CTAB DNA extraction as described above. Trichome DNA samples were run on a 1% agarose gel to assess DNA concentration and quality. For genotyping by Sanger sequencing, 20 ng of trichome DNA was amplified by combining 0.25 µM primer and 1X GoTaq Green Master Mix (Promega, USA) in a 50µl reaction volume. Reactions were subject to 3 minutes at 95˚C, followed by 35 cycles of 30s 95˚C, 30s at reaction-specific annealing temperatures, and 72˚C for

reaction-specific extension times. Reaction specific primer sequences, annealing temperatures and extension times are listed in Table S9. All reactions were subjected to a 5:00 extension at 72˚C. PCR products were purified by SeraMag SpeedBead cleanup and Sanger sequenced with the corresponding forward and reverse amplification primers by GeneWiz (South Plainfield, NJ). Sanger reads were aligned to the predicted DM1-3 amplicon sequence, and positions covering putative L1-specific alleles were identified in each read. At these positions, signal intensity of all four bases was extracted using the sangerseqR (14) and sangeranalyseR (15) packages in R (version 4.3.1). Variant allele frequencies were calculated as the ratio of the channel intensity of the putatively L1-specific allele to the total intensity of all channels at that position. Short read libraries of trichome DNA samples were prepared as previously described (4) (Table S1).

Red Polenta genome assembly and annotation

Genome size estimation
Short reads were used to estimate the genome size of Red Polenta. Jellyfish version 2.2.7 (16) was used to count k-mers (k=31). The genome size and homozygous coverage level was then estimated from the 31-mer histogram output by Jellyfish using GenomeScope 2.0 (17).

De novo assembly
Red Polenta HiFi reads were assembled with hifiasm 0.19.5-r587 (18) using options `--primary --hom-cov 140 -t 32 -l 3`. The primary assembly was subjected to one additional round of haplotig purging with purge_haplotigs (19). Alternate haplotypes from the initial assembly (hifiasm output ".a_ctg.*") and purged primary assembly contigs were concatenated as alternate assembled contigs, but were not used for subsequent read mapping. Assembly completeness and duplication was evaluated using the solanales_odb10 database of BUSCO v.5.3.2 (20) with default parameters.

Repeat and gene model annotation
Transposable elements were identified by the Extensive De-Novo TE Annotator (EDTA) v2.0.0 (21). The non-redundant TE library generated by EDTA was then provided to RepeatMasker v.4.0.7 to mask TEs and simple repeats in the Red Polenta primary assembly. To predict gene models, RNA-seq reads from greenhouse-grown Red Polenta and MF93 leaves and the 20-tissue atlas of tetraploid potato cv. Cooperation88 (22) were aligned to the purged RP primary assembly using HISAT2 v.2.1.0 (23). BRAKER3 was used to annotate genes on the soft-masked genome (24–27), with mapped RNA-seq reads and peptide sequences of potato DM1-3 v6.1 (9), tomato cv. M82 (28) and the Viridiplantae OrthoDB v10 database (20) as input. Within the BRAKER3 pipeline, RNA-seq reads were assembled using StringTie (29), followed by protein alignment and gene model training with GeneMark (30) and AUGUSTUS (31). Transcript- and protein-based gene predictions were combined with TSEBRA (32).

Mutation detection

Desiree mutations
Short reads of Desiree clones from previous studies (1, 33, 34) were downloaded from NCBI SRA and aligned to the Red Polenta primary assembly with BWA mem version 0.7.12r0139 (7). Alignments were processed according to the above section 'Sequence processing and alignment to DM1-3 reference genome'. Raw variants were called with freebayes 1.3.4 (10) and the following parameters: --hwe-priors-off --min-mapping-quality 20 --min-base-quality 20 --genotype-qualities --ploidy 4 --use-best-n-alleles 6. Filters were applied with bcftools 1.19 (11) to remove low-quality variant calls: QUAL > 1, NUMALT==1, MQM>=40, MQMR>=40, |MQM-MQMR| <=15, ≥8x total read coverage in both USDA Urgenta and Depesche, and allele-specific read coverage ≤1x for either the reference or any alternate allele in both USDA Urgenta and Depesche. Subsequent filters were applied in RStudio version 4.4.2. To stringently remove pre-existing variants, sites with one or more reads supporting a putative mutation in USDA Urgenta, IPK Urgenta or Depesche were removed. Mutations in one of four categories were then identified by comparing mutant allele coverage and variant allele frequencies of leaf, root and trichome

samples. L1-specific mutations were called if the mutant allele coverage was 0 in roots, ≥8x with ≥0.125 VAF in trichomes, and ≥1x with VAF < 0.8 in leaves, which identified 1,777 variants. L23-specific mutations were called if the mutant allele coverage was ≥8x with ≥0.125 VAF in roots, exactly zero in trichomes, and ≥1x in leaf with VAF between 0.07 and 0.7, which identified 383 variants. Root-specific mutations were considered if the VAF was < 0.05 in leaves, 0 in trichomes, and >0.02 with ≥3x mutant allele coverage in roots. This step identified 2,090 putative variants with a mean variant allele frequency of 0.11. These variants could represent mericlinal root-specific mutations that we did not consider in this report because they lacked a ready method for cross-validation. Putative mutations in all meristem layers were called if a mutant allele exceeded 8x and 0.125 VAF in leaf, root and trichome samples. This step identified 53 variants that were also present in Desiree clones from CIP and IPK.

As Des-1 tissue samples were taken from greenhouse-grown plants and sequenced as multiplexed libraries, we carried out additional filtering steps to remove biological and index-hopping contaminants. Reads overlapping candidate mutations were aligned to the NCBI nt database with BLASTN (version 2.14.1) (35). Each read was classified as a contaminant if the highest-scoring BLAST hit was not to *S. tuberosum* or another *Solanum* species. Layer-specific mutation calls supported by four or more contaminant reads in any layer-enriched sample were removed.

To estimate the effect of read coverage on mutation calling sensitivity, we used bedtools genomecov to generate per-position coverage histograms of each Desiree sample that was used to identify layer-specific mutations. Overall, fractions of sites with ≤8x total coverage was low (6.33-8.61%) among these samples, suggesting that few mutations fixed within a meristem layer remain undetected due to insufficient read coverage (Fig. S17). Furthermore, the fraction of positions with <8x coverage was highest for Desiree trichomes, suggesting that the degree of L1 bias reported here is a lower bound of the true layer bias. For a summary of filters applied to identify Desiree mutations, see Table S9.

To derive layer-specific Des-1 mutation rates, we estimated the number of sites at which a mutation could have been detected at our filtering thresholds. This was performed in three steps, each of which produced a genomic region file. First, we identified all sites of the Red Polenta primary assembly with ≥8x read coverage, considering only reads with mapping quality ≥20 and base call quality ≥20, for each sample that was considered for Des-1 mutation calling: USDA Urgenta, IPK Depesche, as well as Des-1 leaves, roots and trichomes. Second, we identified all sites in the Red Polenta assembly with a mean mapping quality of less than 40 across all samples, considering only reads with mapping quality ≥ 20 in each sample. Third, we stringently identified putatively heterozygous sites as those with >1x coverage of any two alleles in IPK Depesche and USDA Urgenta. To derive genomic regions that were assayable for mutation calling, we subtracted the mean mapping quality ≤40 regions and putatively heterozygous sites from the ≥8x coverage regions, using bedtools subtract (36). To derive assayable space for distinct genomic features, assayable regions were intersected with regions of each feature using bedtools intersect (36). Divergence times between Desiree clones were inferred from passport data available from the US Potato Genebank and the Potato Pedigree Database (37). Genome-wide and feature-specific mutation rates were then calculated the number of mutations in assayable space per accessible base pairs per year.

Red Polenta mutations

Short reads were aligned to the Red Polenta primary assembly with bwa mem 0.7.12-r1039 (7) with default parameters. Alignments were processed to remove duplicates and mates aligning to different contigs, and to soft-clip the overlapping region of one mate in overlapping paired-end reads as described above (section 'Sequence processing and alignment to DM1-3 reference genome'). Raw variants were called with freebayes 1.3.4 (10) and the following parameters: --hwe-priors-off --min-mapping-quality 20 --min-base-quality 20 --genotype-qualities --ploidy 4 --use-best-n-alleles 6. Initial filters were applied with bcftools 1.19 (11) to remove low-quality variant calls: QUAL > 1, NUMALT==1, MQM>=40, MQMR>=40, |MQM-MQMR| <=15, CIGAR=1x,

≥8 total supporting reads in Urgenta, and reference or alternate allele-specific coverage no greater than 1x in USDA Urgenta. Additional filtering was carried out with RStudio version 2023-06.1+524 (using R version 4.3.2). To remove read alignment artifacts appearing at low levels in each regenerated clone, we adapted a previously described beta binomial filter (38) based on the Shearwater variant caller (39). For each candidate mutation locus, the variant allele-specific and total read depth of each regenerated clone were used to calculate Tarone's Z (40), which estimates goodness of fit to a binomial distribution as a Z-score. The VAF of alignment artifacts was consistently low among many samples, resulting in a good fit to a binomial distribution with little to no overdispersion. However, true mutations are expected to appear at high VAF in some samples but not in others, resulting in the distribution of VAF values among all samples at a site being overdispersed relative to a beta binomial. After inspecting the histogram of site-specific Z scores, we retained variants with score above 8. Layer-specific mutations were called if a mutant allele was detected at ≥1x coverage in Red Polenta leaves, at ≥0.125 VAF in four or more regenerated clones of one type (carrying either tr8-7 or hap8), and at ≤0.125 VAF in all clones of the other type. For L1-specific mutations, we further required that mutant alleles were strictly absent from roots, present at VAF <0.8 in leaves, and present at VAF >0.01 in L1 regenerants MF43 and MF105. For L2,3-specific mutations, we further required root VAF >0.05, leaf VAF between 0.07 and 0.9, and VAF<0.01 in regenerants MF43 and MF105. Candidate root-specific mutations were called if root VAF>0.02, leaf VAF<0.05 and VAF<0.01 in regenerants MF105 and MF43 and subject to BLASTn contaminant removal, as described above. To account for short read alignment artifacts, we also required that mutations were detected in long-read alignments of Red Polenta and layer-specific regenerants. Long reads were aligned to the primary assembly with pbmm2 version 1.10.0 with parameters --preset CCS. All forms of secondary and supplementary alignment were removed with samtools view -F 3840. Variants were called with freebayes 1.3.4 (10), restricting variant calling to only the candidate mutations retained after filtering of short read-based candidate mutations. After manually inspecting long- and short-read alignments of all remaining candidate mutations in IGV, we removed 73 candidate mutations that likely corresponded to read alignment artifacts: 38 candidates lacked long read support for the mutant allele in Red Polenta and all regenerated lines, another 23 candidates were present at ≥5% VAF in long read alignments of Red Polenta and all regenerated lines, and an additional 12 candidates were adjacent to homopolymers. For a summary of filters applied to identify Red Polenta mutations, see Table S9.

To derive assayable space for Red Polenta mutation calling, we used a three-step filter similar to our approach for Des-1. First, to identify sufficiently covered positions of the Red Polenta primary assembly, we identified all sites with ≥8x total coverage in each of the following samples: USDA Urgenta, four or more L1-specific regenerants, and four or more L23-specific regenerants, in each sample counting reads with mapping quality ≥ 20 and base quality ≥ 20. Coverage analysis indicated that >91% of positions are covered at least 8 times in all samples, except for L1 regenerant MF_113 (Fig. S18). Second, to remove sites with poor mapping quality across all samples, we calculated the average read mapping quality per site from read alignments of both Urgenta clones (USDA and IPK), Red Polenta leaves and roots, Tricoli-RP leaves, stem explant regenerants D3, D59 and D140, and all protoplast-regenerated lines, and removed sites with mean mapping quality < 40. Third, to strictly filter out variants that pre-existed in Urgenta, we removed sites with ≥1x coverage both the reference allele and any alternate allele in both USDA Urgenta and IPK Urgenta.

To determine Red Polenta L2,3 mutation presence in L2, we reanalyzed previously described short read data of 288 Red Polenta offspring, including dihaploids, tetraploid selfs, and Red Polenta x IvP48 hybrids (5, 41). Reads were processed and aligned to the RP primary assembly as described above. Loci with <8x aggregate coverage across the entire population were not considered. For the remaining loci, we classified reads overlapping L1-specific or L2,3-specific mutations as ancestral or derived. For each layer category (L1 or L2,3), we then reported the proportion of reads supporting the derived allele across all layer-specific mutations.

Mutation rate in regenerants
Mutations that were specific to single protoplast-regenerated lines or RP roots were identified from the same set of raw variants generated for the identification of layer-specific mutation calls, and subject to the same first-pass filters for variant quality, biallelism, mean mapping quality across all samples, ≥8x USDA Urgenta read coverage, and ≤1x mutant allele coverage, as described above. We then filtered to retain sites for which a mutation was detected in a single regenerated line at ≥8x mutant allele coverage. For each sample, false positives due to biological contaminants or index-hopping artifacts were identified via the blastn-based approach described above, and sites exhibiting more than one contaminant read in a sample were removed. After each variety was created by a sexual process, it underwent vegetative growth for different times and potentially different conditions (field vs. tissue culture propagation). Tracing the propagation history of each clone would be impossible. We used a “per year" rate. Since we focused on fixed mutations, the calculated rate is based on clone age, a biologically relevant measure and serves the purpose of evaluation and comparison.

Regenerated explant layer of origin
To determine the layer from which regenerated Desiree stem explants originated, short reads overlapping each Desiree L1- or L2,3-specific mutation were binned, and the fraction of reads supporting the L1-specific allele were determined for each stem regenerant. We analyzed L1- and L2,3-specific mutant allele sets separately. To assign origin from L1, we set a 10% read support threshold: if ≥10% of reads at all loci with L1-specific mutations matched the mutant allele, we concluded that the regenerant was partially or completely derived from L1, otherwise, the regenerant was not derived from L1. To assign origin to L2,3, we used a 10% read support threshold using only reads that overlapped L2,3-specific mutations. If read support for L1- or L2,3-specific alleles both exceeded 10% for a regenerant, we concluded that the regenerant was derived from all three layers. To determine the layer composition of Red Polenta regenerants D3, D59 and D140, we used a similar approach, except that L1-specific alleles at hap8-1 specific loci (rather than L1- and L2,3-specific mutations) were counted.

Figures

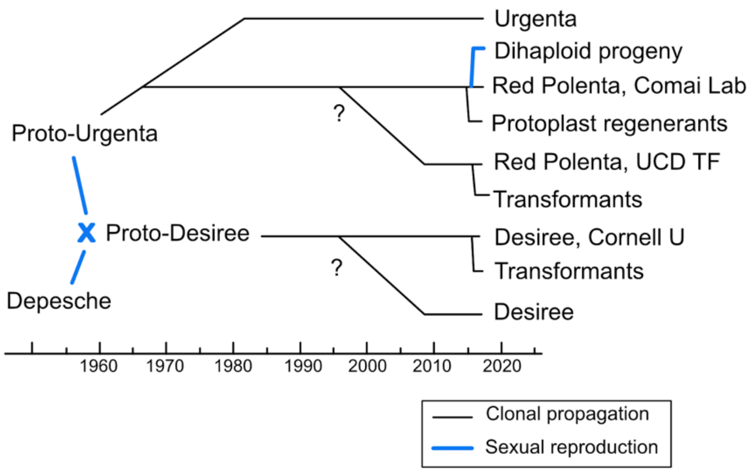

Fig. S1. Experimental material. Genomic analysis entailed ~35 years of divergence for Desiree and 60 years for Red Polenta. The marked dates (?) for subclone formation are approximate.

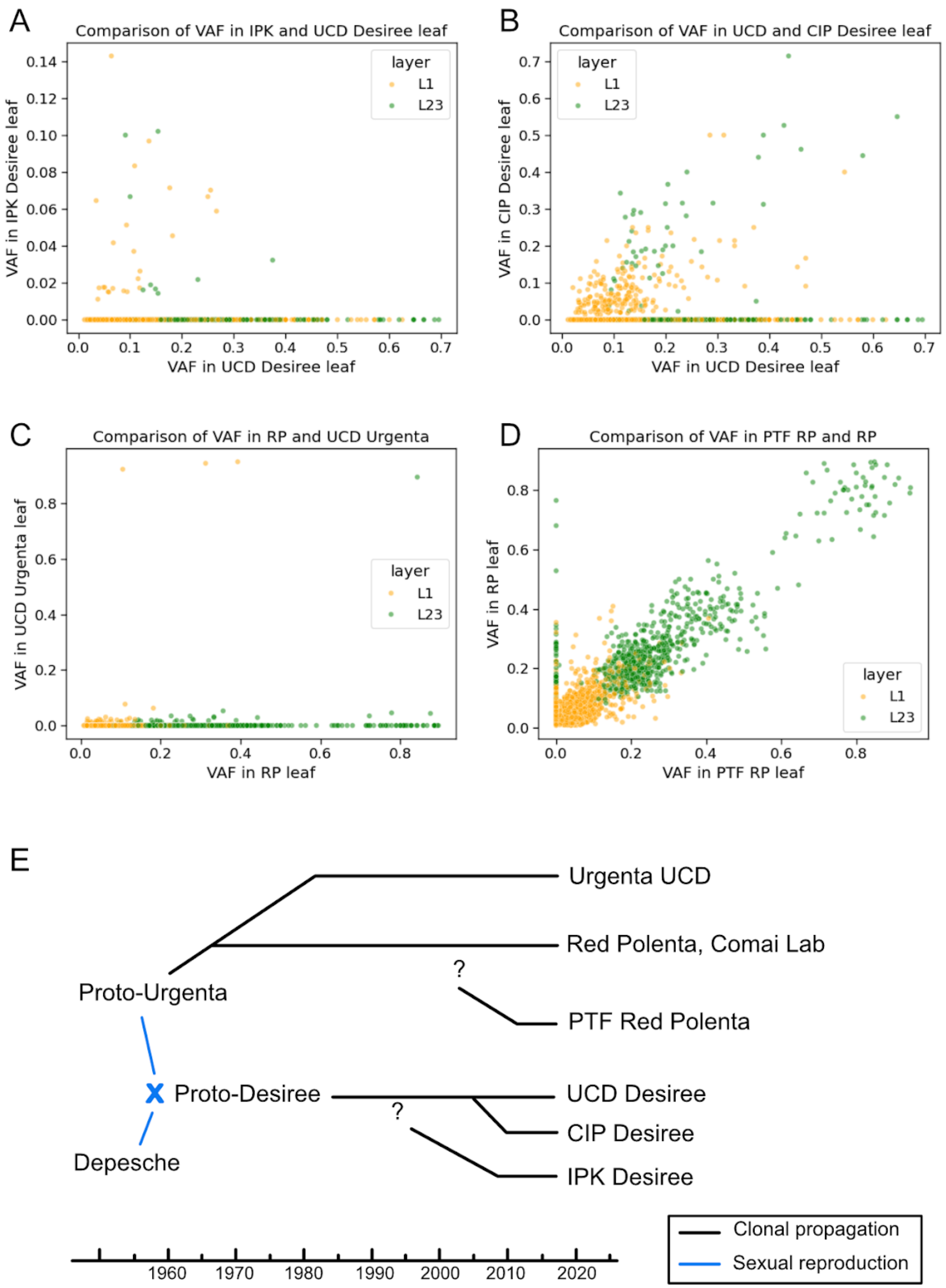

Fig. S2. Shared SNP in the Desiree and Red Polenta lineages. A-B. Of the 1785 and 384 L1 and L23 mutations identified in the UC Davis clone of Desiree, only 33 are also present in the IPK clone of Desiree (A) and 252 are also present in the Centro National de la Papa (CIP) clone of Desiree (B). C-D. Of the 1856 L1 and L23 mutations identified in the UC Davis clone of Red

Polenta, only 4 mutations are shared with Urgenta (the clonal parent of Red Polenta) and 1715 with another UC Davis clone held at the Plant Transformation Facility. The time since acquisition of Red Polenta by the PTF is unknown, but estimated to be decades. E. Family tree of the potato clones used in this analysis.

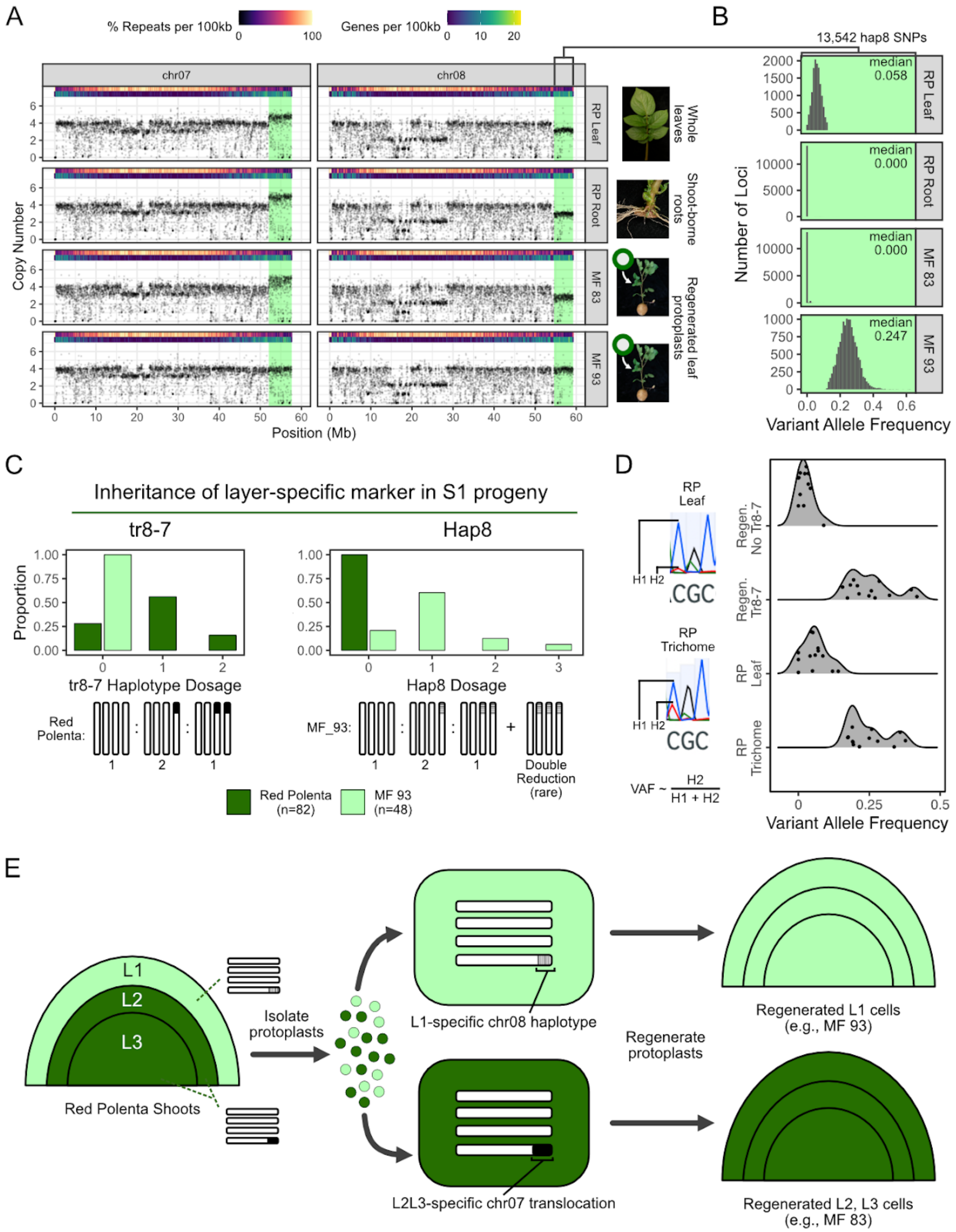

Fig. S3. Genomic signatures of periclinal chimerism in potato. A translocation in Red Polenta demonstrates the analysis of layer-specific polymorphism, validating the extension of the connected methods to mutation analysis. A) Read coverage plots display five copies of the tip of chromosome 7 and 3 copies of the tip of chromosome 8, indicating the presence of a tr8-7 translocation in leaf and root cells of Red Polenta (RP). The same signal is present in regenerant MF83 but absent in regenerant MF93. B) Variant Allele Frequency (VAF) for 13,542 SNPs spanning the tip of chromosome 8. One haplotype, called H08-1, is substoichiometric in Red Polenta leaves because it is only present in the L1 layer. It is absent in Red Polenta roots

because they are formed preponderantly from the L3. It is also absent in L2,3 regenerant MF83, and present at the expected simplex VAF in L1 regenerant MF93. C) Inheritance of tr8-7 and haplotype H08-1. Self-fertilized offspring of Red Polenta (n=82) exhibited Mendelian segregation with respect to tr8-7 but complete absence of haplotype H08-1. On the other hand, L1 regenerant MF93 and its offsprings completely lacked tr8-7 but haplotype H08-1 segregated as expected, including a few cases of double reduction D) Selected SNPs from haplotype H08-1 were PCR amplified and scored by Sanger DNA sequencing. The VAF illustrates the normal simplex state in an L1 regenerant, its absence in an L2,3 regenerant, the low VAF resulting from minority state in Red Polenta leaves, and the VAF corresponding to simplex allele dosage in Red Polenta trichomes. E) Model of chimerism and isolation of clonal derivatives through protoplast regeneration. Red Polenta is a periclinal chimera in which the L1 layer is wild-type with respect to tr8-7 and has a unique, ancestral chromosome 8 haplotype. The L2 and L3 layers are mutant and share translocation tr8-7.

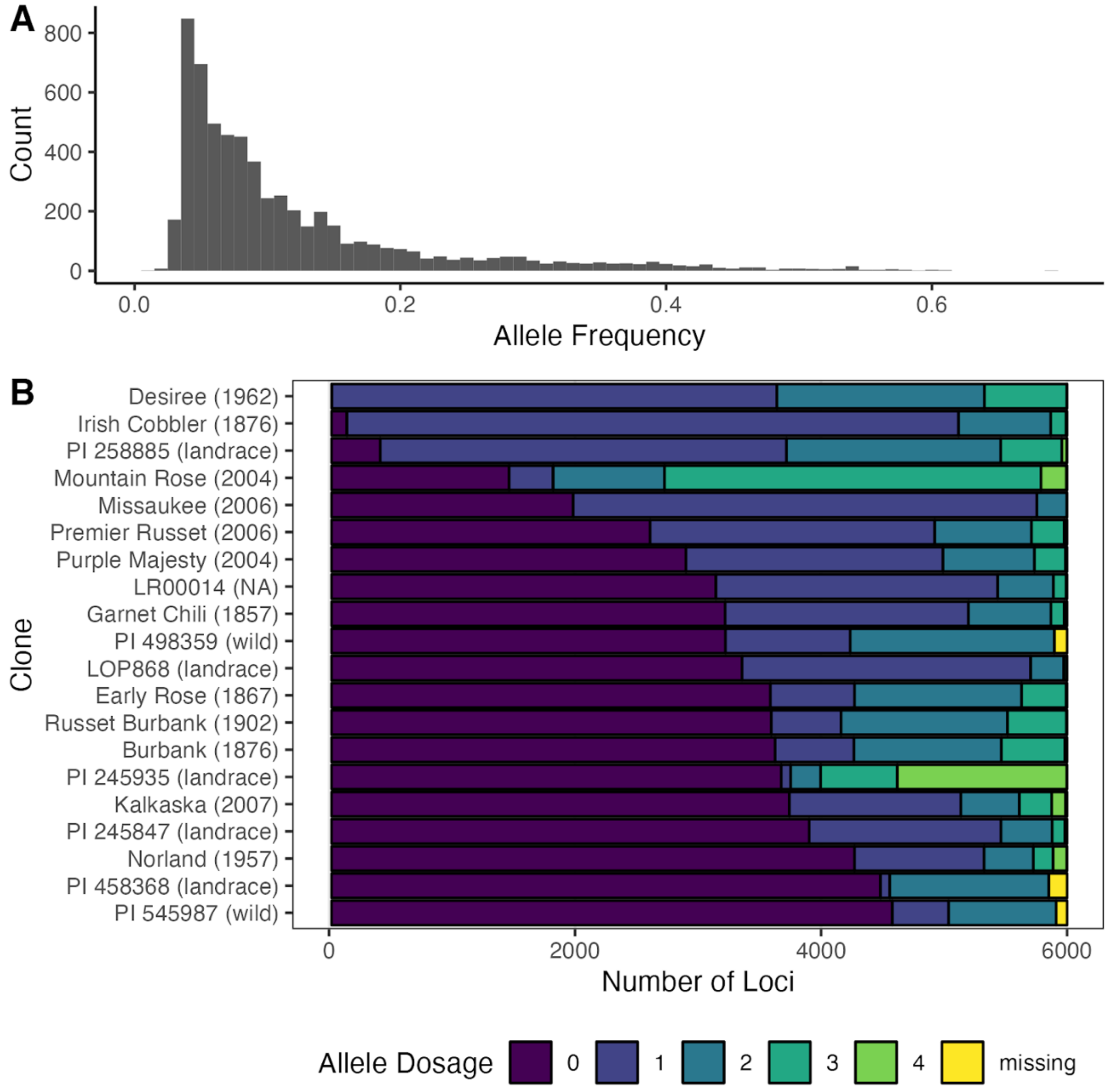

Fig. S4. SNP alleles associated with tr8-7 absence in Red Polenta are common among potato varieties. A) Frequency spectrum of approximately 6,000 SNP alleles associated with tr8-7 absence in Red Polenta among a potato diversity panel. Allele frequencies greater than zero indicate appearance of these alleles in other potato varieties. B) Bar plot illustrating the 20 individuals with the greatest extent of allele sharing among the diversity panel. Bar color indicates allele dosage. Allele sharing with landraces, wild species and varieties released before Urgenta in 1951 indicate reappearance of an ancestral haplotype. Pedigree release dates from (37).

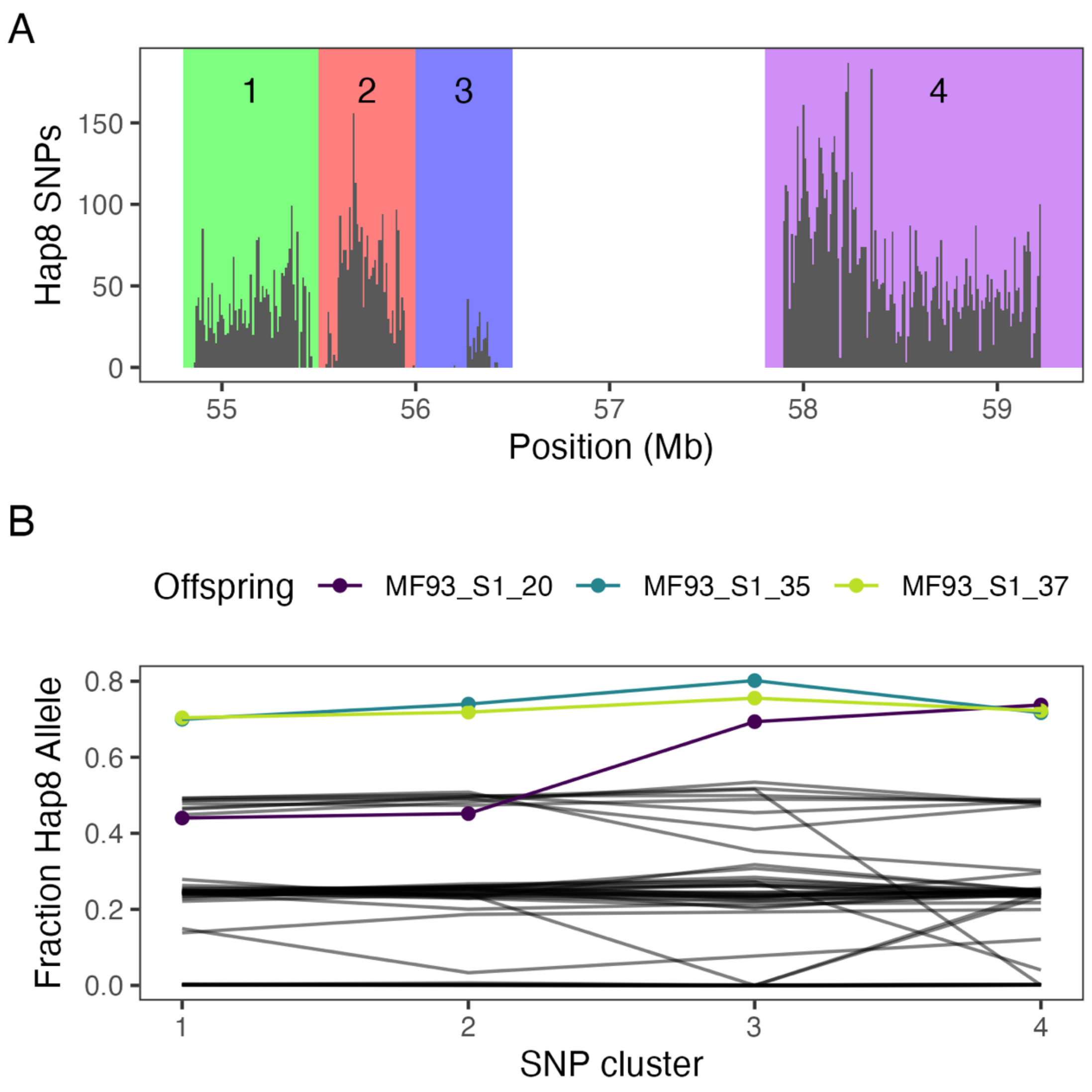

Fig. S5. Evidence of double reduction in MF93 S1 progeny. A) Histogram of hap8 SNP loci along the distal arm of chromosome 8. Bars correspond to the number of hap8 SNP loci in non-overlapping 10kb windows. For hap8 dosage analysis, SNPs along hap8 were binned into four regions. B) Fraction of hap8 allele by region in MF93 S1 offspring. Three offspring with hap8 dosage exceeding 2 are shown as lines with unique colors; all other offspring are shown as black lines. Locations of crossovers involving hap8 are indicated by a shift in hap8 allele fraction between adjacent regions. Fractions exceeding 0.6 across all four clusters indicate a crossover double reduction between region 1 and the centromere. A change in fraction from approximately 0.5 to approximately 0.75 indicates double reduction due to a crossover between regions 2 and 3.

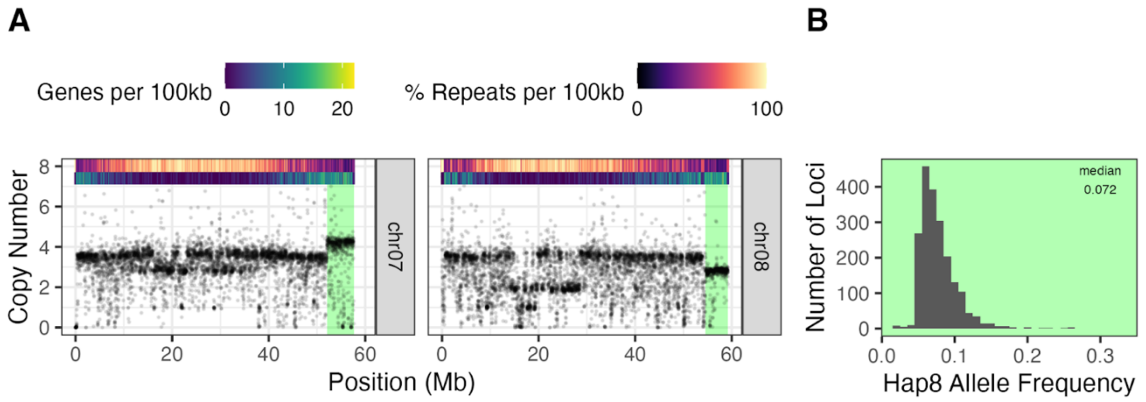

Fig. S6. A second Red Polenta holding exhibits the tr8-7 periclinal chimerism. A) Read depth of non-overlapping 10kb windows on chromosomes 7 and 8 reveals the tr8-7 pattern. B) Histogram of hap8-specific allele frequencies, bin size 0.01.

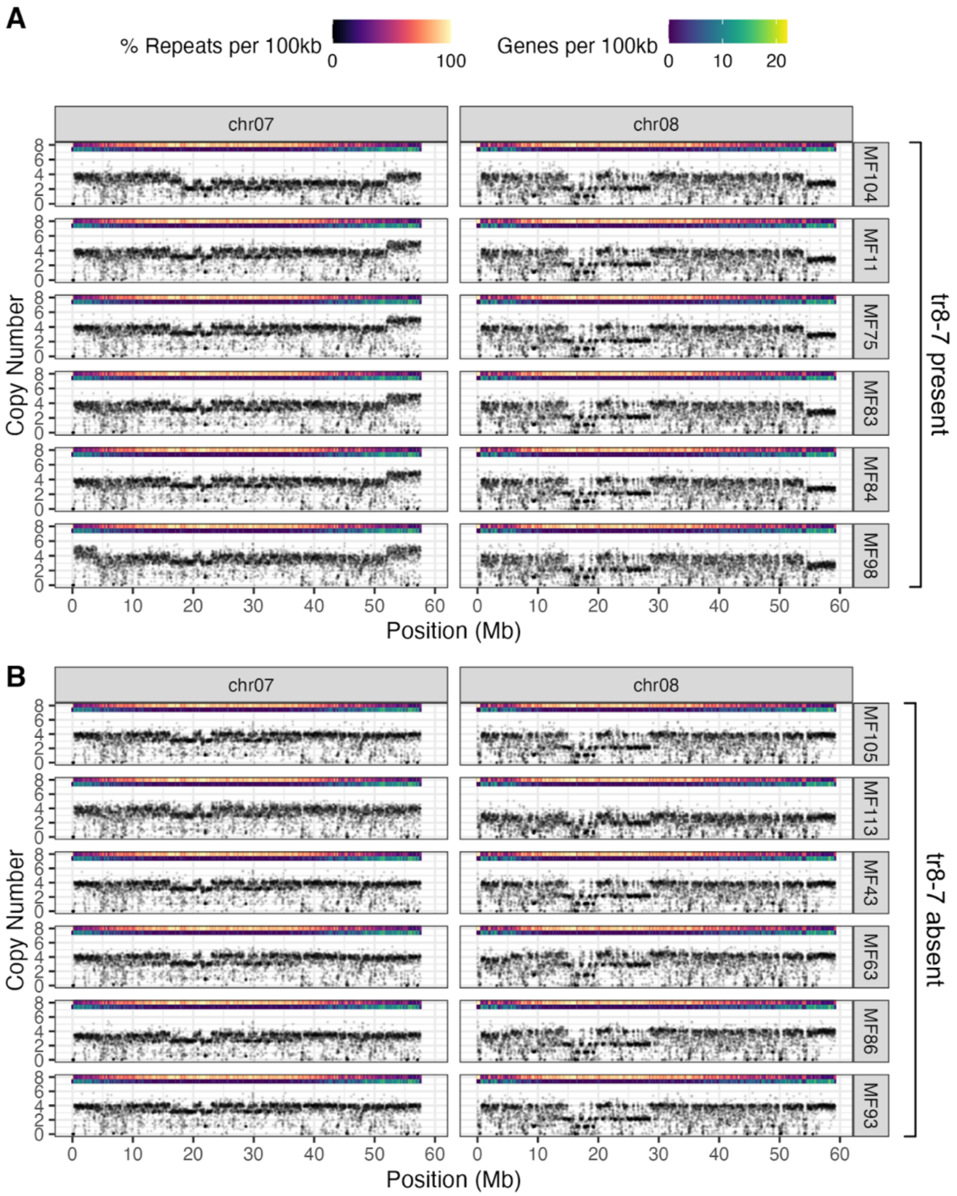

Fig. S7. Read depth analysis of regenerated leaf protoplasts of Red Polenta. Each data point corresponds to the median read depth of a non-overlapping 10kb window of the DM1-3 v6.1 reference genome, with only chromosomes 7 and 8 shown. A) Six regenerated lines exhibited tr8-7 after regeneration. B) Six regenerated lines lacked tr8-7 after regeneration.

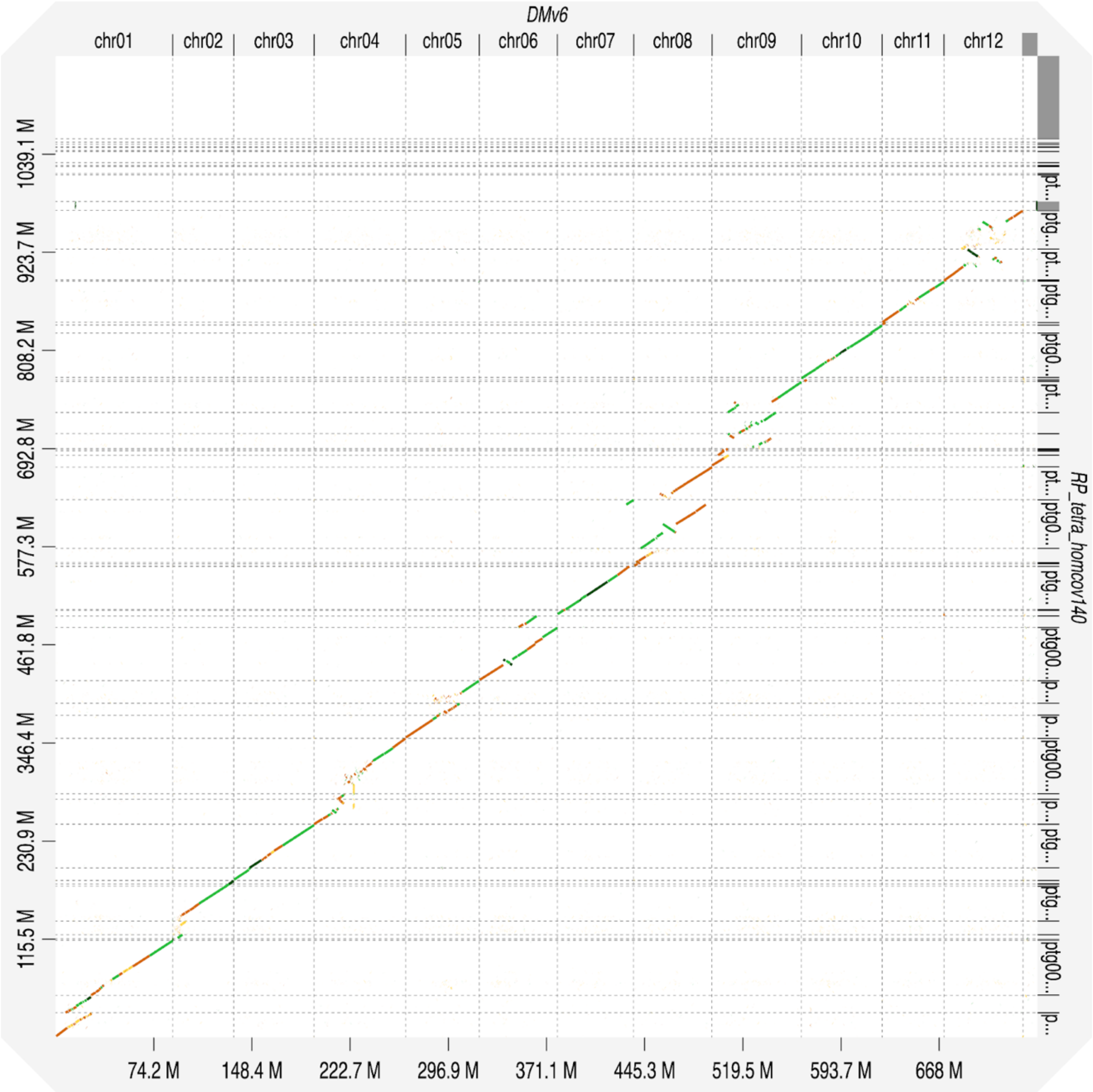

Fig. S8. Attributes of the Red Polenta primary assembly. A) Dotplot showing alignments to reference assembly DM1-3 v6.1, showing retention of allelic regions in the RP assembly.

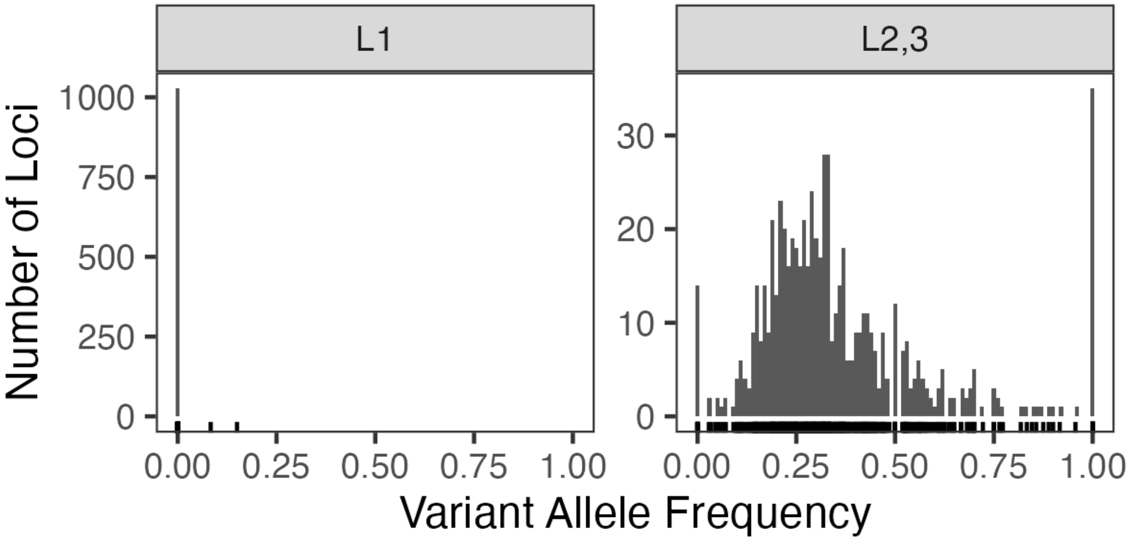

Fig. S9. Transmission of Red Polenta SNP to progeny. The inheritance of Red Polenta SNP was examined according to their layer location. Both in dihaploids and selfed progeny, L1 SNP were not inherited. L2,3 SNP, on the other hand, were present in both progeny set according to inheritance expectations for simplex SNP and displayed a mean VAF of 0.25, while in Red Polenta they display a mean VAF of 0.19-0.20.

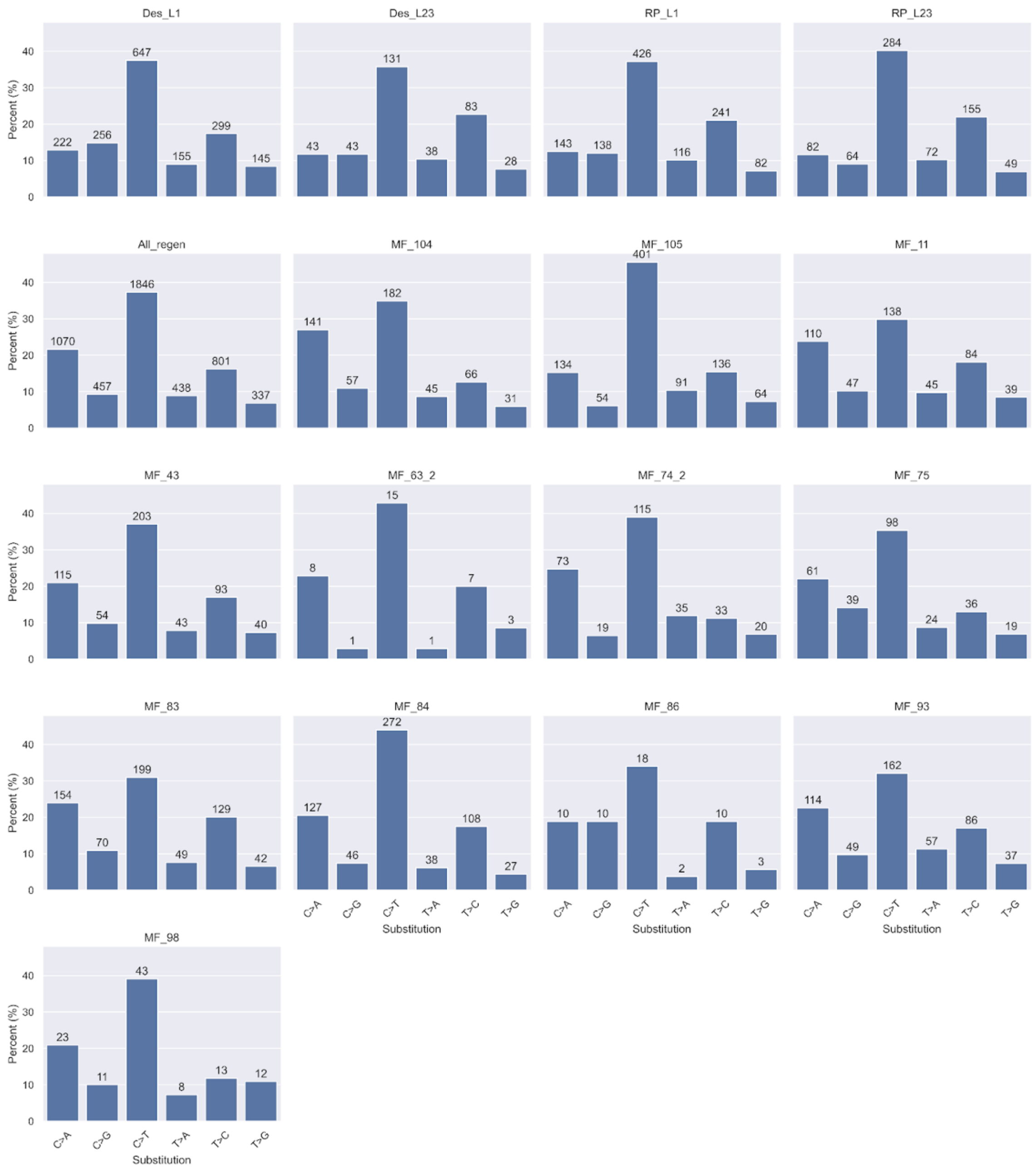

Fig. S10. Single base substitutions. Each panel displays percent (Y axis) and counts (top of each bar) for Desiree L1 and L2,3, Red Polenta L1 and L2,3, and for all single cell regenerants except MF113, which was excluded due to low sequencing coverage.

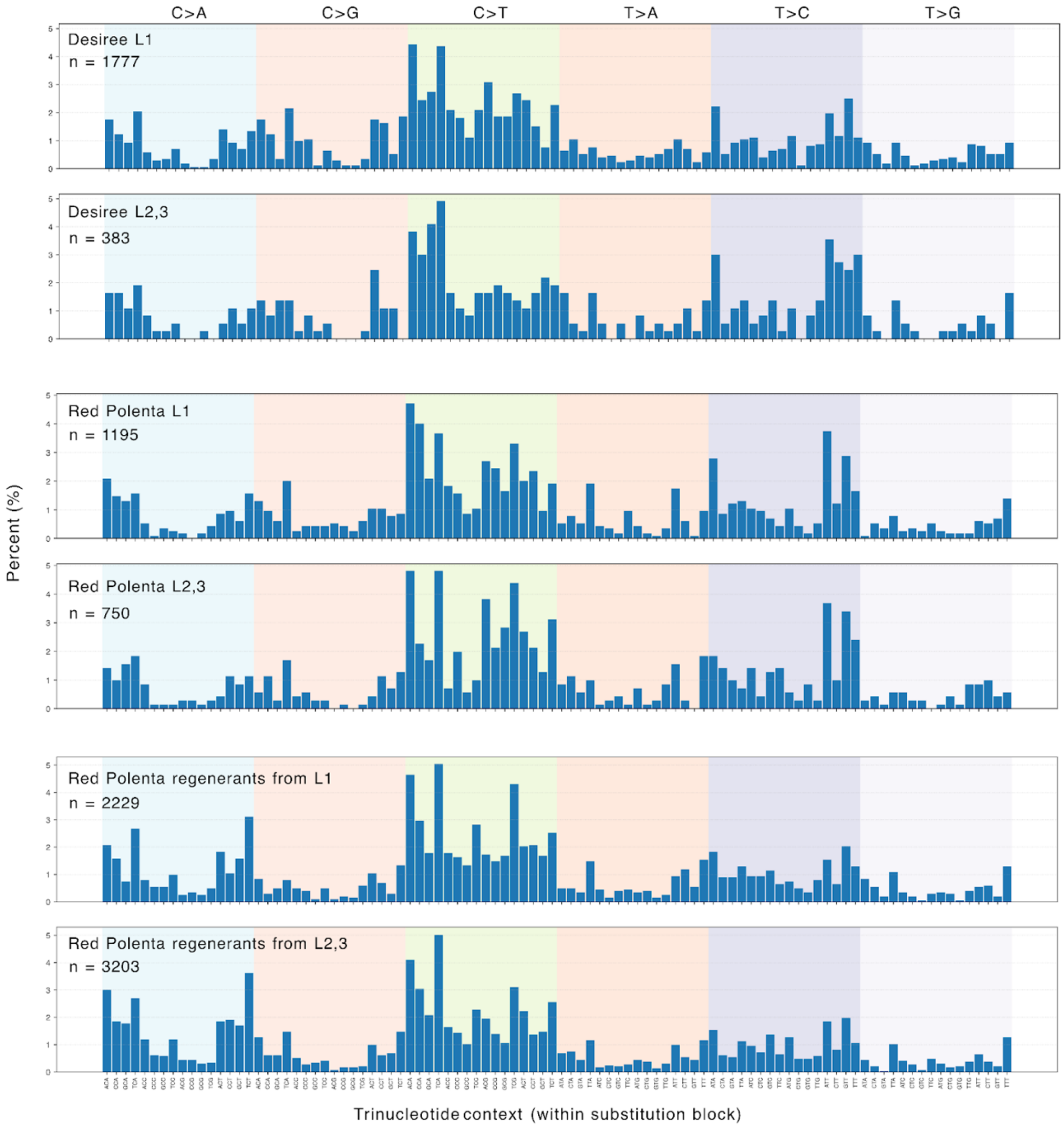

Fig. S11. Raw trinucleotide profiles of SNV. Layer-specific profiles for RP, Desiree, and all regenerants (cumulative).

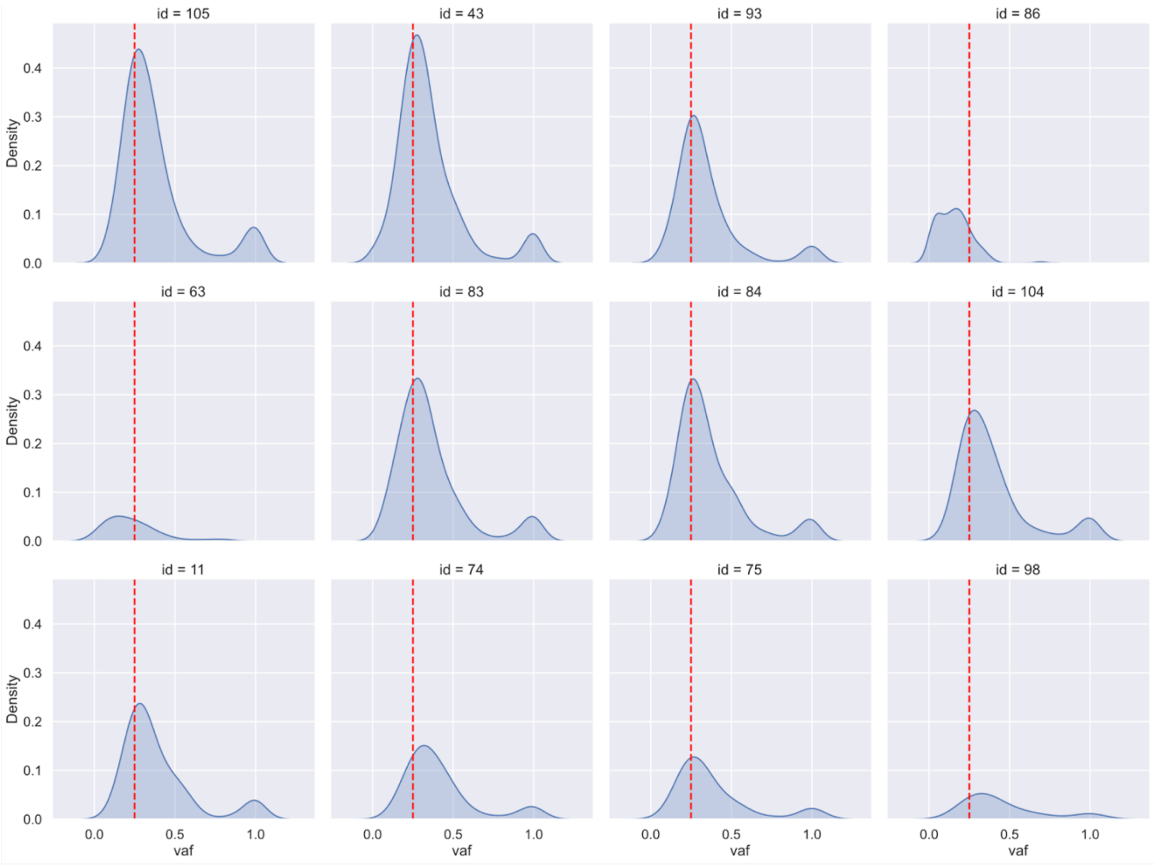

Fig. S12. Variant allele frequency of mutations accumulated during regeneration. Each panel represents the distribution of VAF in a single regenerant. The red hatched line marks VAF = 0.25, which is expected for a simplex mutation in a tetraploid.

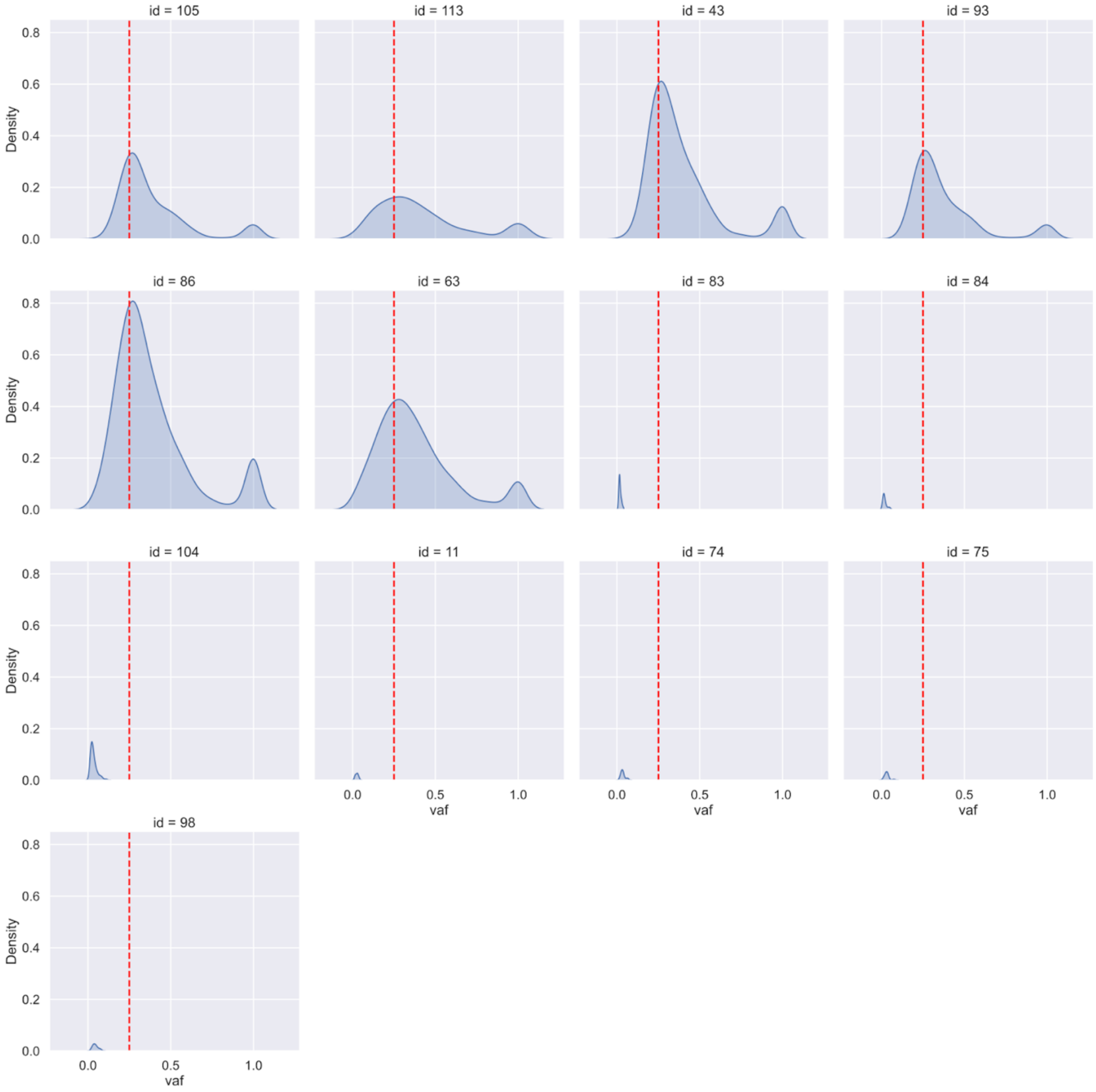

Fig. S13. Variant allele frequency of regenerants' mutations fixed in the L1 of Red Polenta. Each panel represents the distribution of VAF in a single regenerant. The red hatched line marks VAF = 0.25, which is expected for a simplex mutation in a tetraploid.

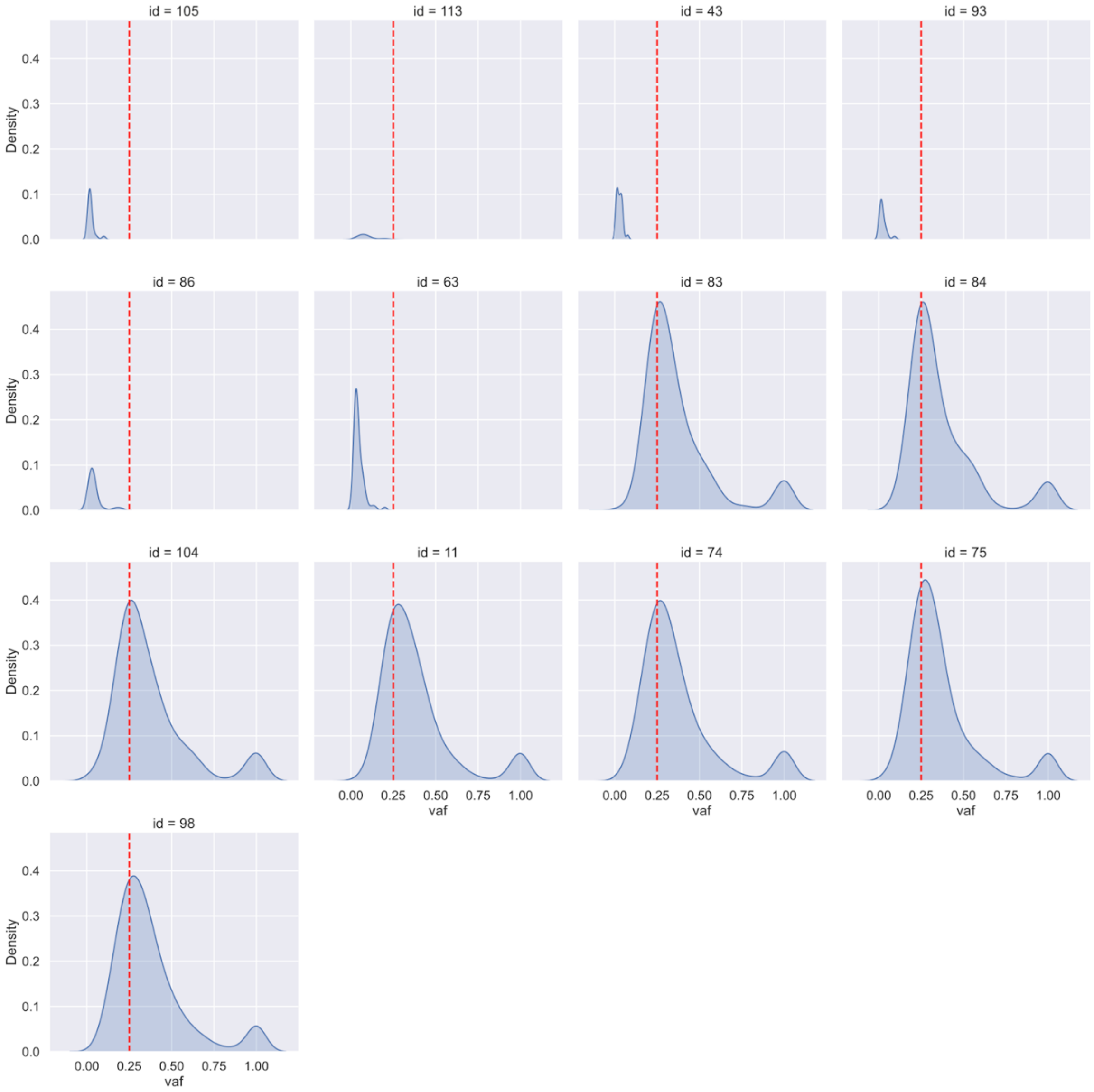

Fig. S14. Variant allele frequency of regenerants' mutations fixed in the L2,3 of Red Polenta. Each panel represents the distribution of VAF in a single regenerant. The red hatched line marks VAF = 0.25, which is expected for a simplex mutation in a tetraploid.

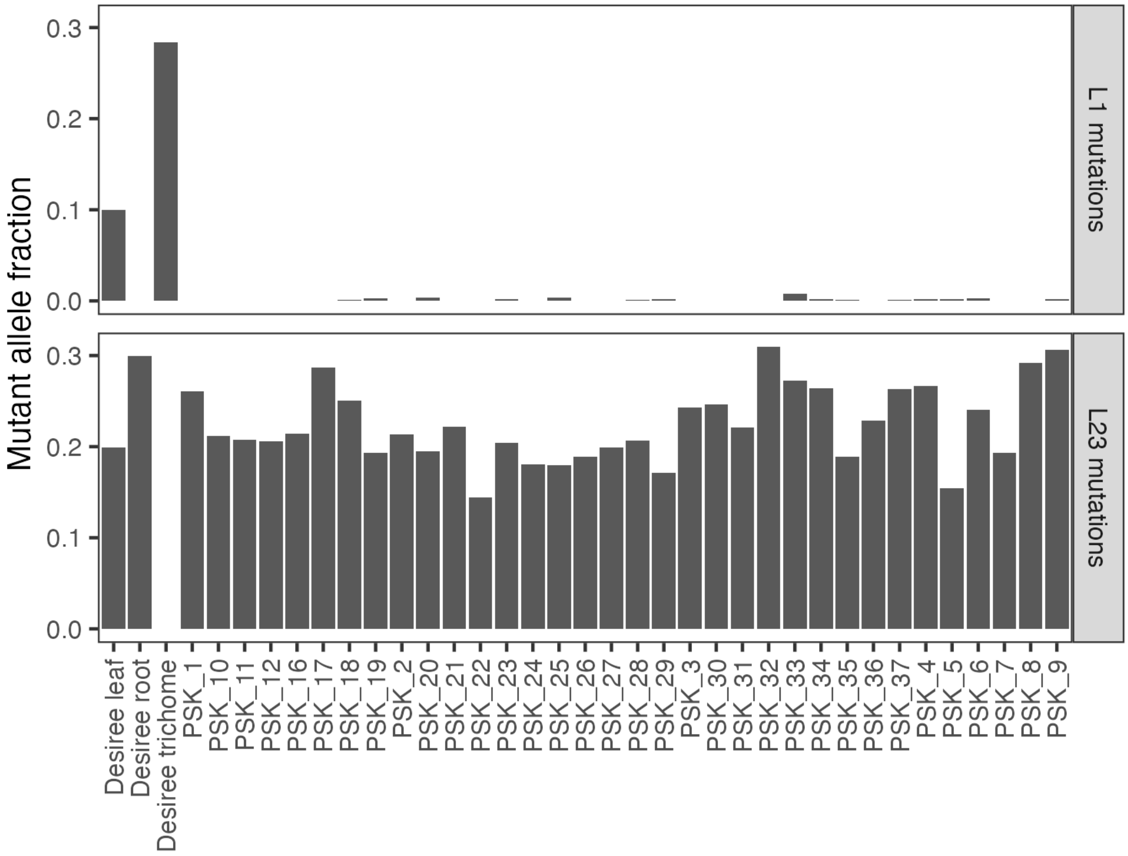

Fig. S15. Regenerated stem explants of Des-1 originated from L2,3. Low-coverage sequencing of 34 independently regenerated Des-1 plants from stem explants were aligned to the RP assembly. Reads overlapping L1-specific or L2,3-specific were assigned to ancestral or derived alleles. Read counts were aggregated by locus, and the fraction of reads supporting the layer-specific mutant allele was used to determine whether each stem regenerant originated from the L1, the L2,3 or from all layers.

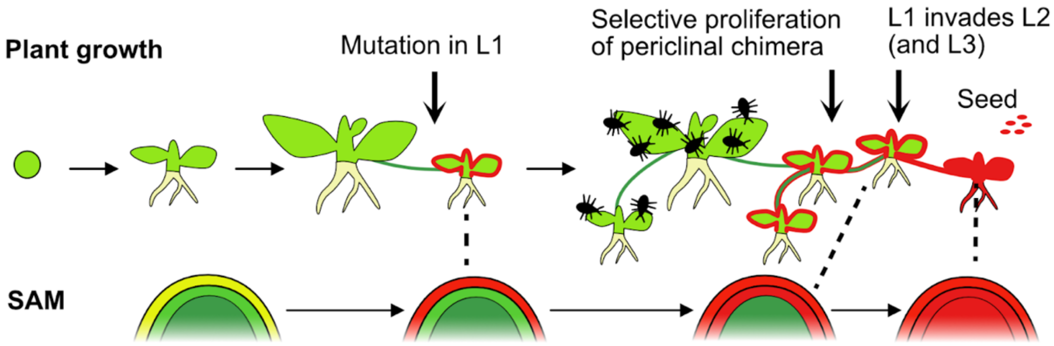

Fig. S16. Hypothetical example of L1-dependent selection. A mutation in the L1 protects the epidermis from a herbivore causing biased growth of the mutant plant part and clonal expansion. Eventually, a rare L1->L2 invasion introduces the mutation in the sexual cycle capturing the novelty.

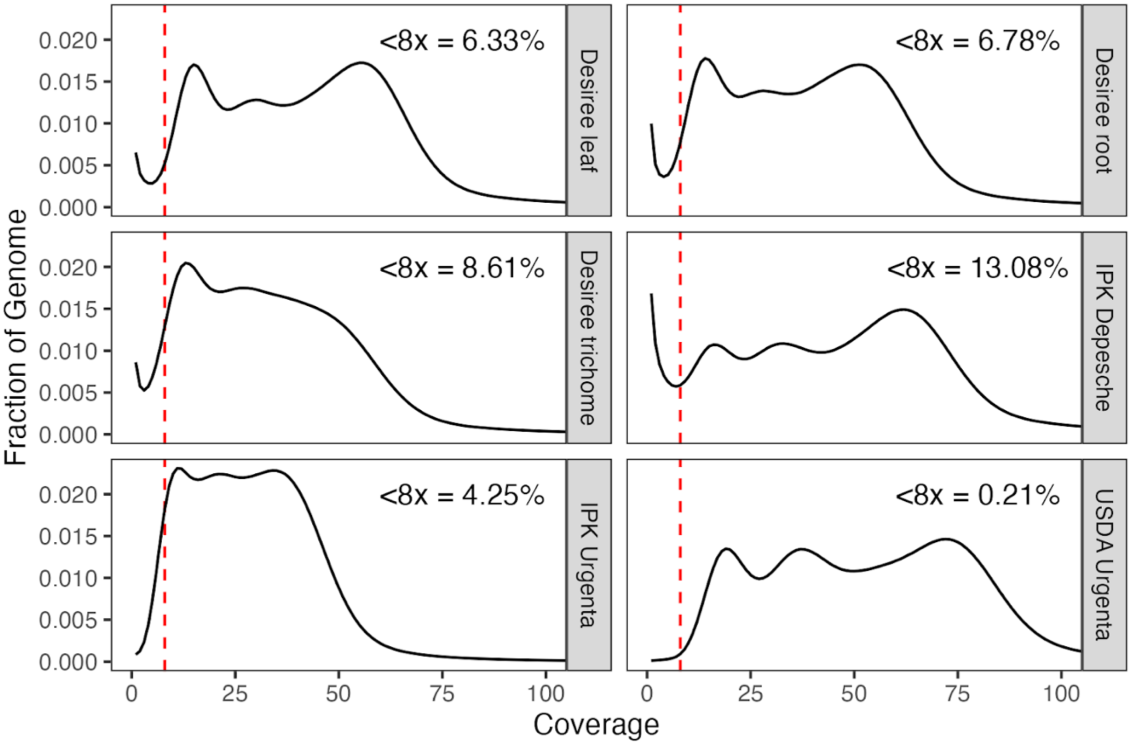

Fig. S17. Coverage analysis of Desiree samples used for mutation calling. Read coverage histograms indicating the percentage of the genome covered by a specific number of reads. The red dashed line in each panel indicates 8x coverage. Positions covered by fewer than eight reads were excluded from mutation calling.

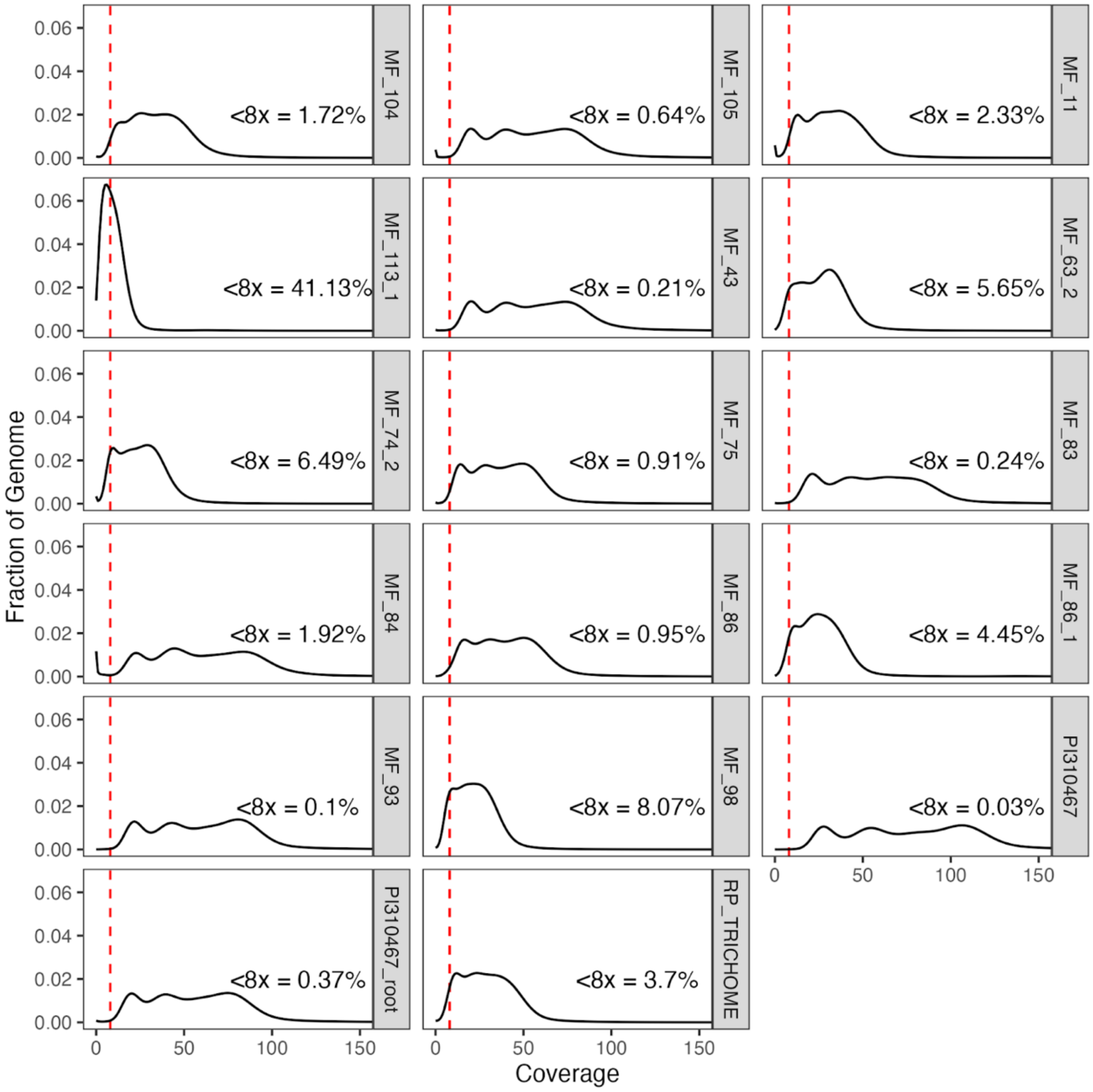

Fig. S18. Coverage analysis of Red Polenta samples used for mutation calling. Read coverage histograms indicating the percentage of the genome covered by a specific number of reads. The red dashed line in each panel indicates 8x coverage. Positions covered by fewer than eight reads were excluded from mutation calling. Due to low coverage, MF113 was excluded from analysis.

Tables

Table S1. Sequencing library information
10.6084/m9.figshare.29975758

Table S2. Counts of mutation properties and related statistical analyses
10.6084/m9.figshare.30183067

Table S3. Assembly summary

	RP primary	RP purged
**Assembly Size**	1154595727	983369830
**Contigs**	2474	365
**Contig N50**	38513535	45453008
**Genome BUSCO**	C:98.5%[S:80.3%,D:18.2%],F:0.1%,M:1.4%,n:5950	C:98.3%[S:85.2%,D:13.1%],F:0.1%,M:1.6%,n:5950
**Repeat content**	0.6419111449	0.6718998711
**Gene count**	46046	38417
**Mean Gene Length**	4027.387	4436.042
**Transcript Count**	55683	47108
**Mean transcript length**	4669.078	5105.117
**Mean exon length**	256.5949	244.4221
**Mean exon count per gene**	4.128763	4.526303
**Protein BUSCO**	C:99.3%[S:80.9%,D:18.4%],F:0.1%,M:0.6%,n:5950	C:99.0%[S:85.7%,D:13.3%],F:0.1%,M:0.9%,n:5950

Supplementary Tables S4-8 can be downloaded at https://figshare.com/s/2e8aa1d7f72d10c1fb9d

Supplementary Table S4
Des_layer_mutations_final_with_trinucl.tsv, 2.64 MB

Supplementary Table S5
RP_layer_mutations_final_with_trinucl.tsv, 4.07 MB

Supplementary Table S6
RP_samplespecific_final_with_trinucl.tsv, 5.22 MB

Supplementary Table S7
Amundson_Layer_Mutation_Trinucleotide_Counts_S6.xlsx, 13.02 KB

Supplementary Table S8
Desiree_L1_specific.xlsx, 30.24 KB

Table S9. Filtering strategy for mutation discovery

Desiree Filtering Steps and Outcomes

Filter applied	Number of variants
None (raw variants)	36,338,067
QUAL>1<br>NUMALT==1<br>MQM >= 40<br>MQMR >= 40<br>abs(MQM-MQMR) <= 15<br>Urgenta DP >= 8<br>Urgenta RO <= 1 or Urgenta AO <= 1<br>Depesche RO <= 1 or Depesche AO <= 1	907,589
Urgenta and Depesche homozygous genotype calls	177,486
Depesche DP >= 8	153,301
Depesche VAF == 0	112,692
Urgenta VAF == 0	78,827
IPK Urgenta VAF == 0	41,111
**L1-specific group**	
UCD Desiree root VAF == 0	17,708
UCD Desiree trichome VAF >= 0.125	5,270
UCD Desiree trichome mutant allele coverage >= 8	2,081
UCD Desiree leaf VAF > 0	1,785
UCD Desiree trichome coverage >= 8	1,777
**L23-specific group**	
UCD Desiree trichome VAF == 0	20,623
UCD Desiree root VAF >= 0.125	4,932
UCD Desiree leaf VAF > 0	1,462
UCD Desiree root mutant allele coverage >= 8	384
UCD Desiree trichome coverage >= 8	383
**Root-specific group**	
UCD Desiree trichome VAF == 0	20,623
UCD Desiree trichome DP >= 8	16,095
UCD Desiree leaf VAF == 0	12,093
UCD Desiree leaf coverage >= 8	11,636
UCD Desiree root mutant allele coverage >= 8	52
**All-layer group**	
UCD Desiree root VAF >= 0.125	10,009
UCD Desiree leaf VAF >= 0.125	2,842

UCD Desiree trichome VAF >= 0.125	876
UCD Desiree trichome mutant allele coverage >= 8	65
UCD Desiree root mutant allele coverage >= 8	55

Red Polenta Filtering Steps and Outcomes

**Filter applied**	**Number of variants**
none (raw variants)	29,533,706
QUAL > 1	20,395,800
NUMALT==1	18,105,106
MQM>=40	12,720,095
MQMR>=40<br>abs(MQM-MQMR)<=15<br>URGENTA_DP >=8<br>URGENTA_AO <=1 OR URGENTA_RO <=1	379,654
Compare protoplast regenerants<br>L1 if:<br>• ≥4 regenerants without tr8-7 ≥0.125 VAF and ≥8x mutant allele coverage<br>• All regenerants with tr8-7 <0.125 VAF and ≥8x mutant allele coverage<br>L23 if:<br>• All regenerants without tr8-7 <0.125 VAF and ≥8x mutant allele coverage<br>• ≥4 regenerants with tr8-7 ≥0.125 VAF and ≥8x mutant allele coverage	3,298 (1,498 L1: 1,800 L23)
Tarone Z >= 8	2,090 (1,296 L1: 794 L23)
RP leaf VAF > 0	2,051 (1,261 L1 : 790 L23)
Long read alignment concordance	1,945 (1,195 L1 : 750 L23)

SI References

1. K. R. Amundson, I. M. Henry, L. Comai, The United States Potato Genebank Holding of cv. Desiree is a Somatic Mutant of cv. Urgenta. *Am. J. Potato Res.* **100**, 27–38 (2023).
2. M. Fossi, K. Amundson, S. Kuppu, A. Britt, L. Comai, Regeneration of Solanum tuberosum Plants from Protoplasts Induces Widespread Genome Instability. *Plant Physiol.* **180**, 78–86 (2019).
3. P. W. Inglis, M. de C. R. Pappas, L. V. Resende, D. Grattapaglia, Fast and inexpensive protocols for consistent extraction of high quality DNA and RNA from challenging plant and fungal samples for high-throughput SNP genotyping and sequencing applications. *PLoS One* **13**, e0206085 (2018).
4. K. R. Amundson, *et al.*, Genomic Outcomes of Haploid Induction Crosses in Potato (Solanum tuberosum L.). *Genetics* **214**, 369–380 (2020).
5. L. Comai, *et al.*, LD-CNV: rapid and simple discovery of chromosomal translocations using linkage disequilibrium between copy number variable loci. *Genetics* **219** (2021).
6. M. Martin, Cutadapt removes adapter sequences from high-throughput sequencing reads. *EMBnet.journal* **17**, 10–12 (2011).
7. H. Li, Aligning sequence reads, clone sequences and assembly contigs with BWA-MEM. *arXiv [q-bio.GN]* (2013).
8. G. Jun, M. K. Wing, G. R. Abecasis, H. M. Kang, An efficient and scalable analysis framework for variant extraction and refinement from population-scale DNA sequence data. *Genome Res.* **25**, 918–925 (2015).
9. G. M. Pham, *et al.*, Construction of a chromosome-scale long-read reference genome assembly for potato. *Gigascience* **9** (2020).
10. E. Garrison, G. Marth, Haplotype-based variant detection from short-read sequencing. *arXiv [q-bio.GN]* (2012).
11. P. Danecek, *et al.*, Twelve years of SAMtools and BCFtools. *Gigascience* **10** (2021).
12. M. A. Hardigan, *et al.*, Genome diversity of tuber-bearing Solanum uncovers complex evolutionary history and targets of domestication in the cultivated potato. *Proc. Natl. Acad. Sci. U. S. A.* **114**, E9999–E10008 (2017).
13. I. M. Henry, M. S. Zinkgraf, A. T. Groover, L. Comai, A System for Dosage-Based Functional Genomics in Poplar. *Plant Cell* **27**, 2370–2383 (2015).
14. J. T. Hill, *et al.*, Poly peak parser: Method and software for identification of unknown indels using sanger sequencing of polymerase chain reaction products. *Dev. Dyn.* **243**, 1632–1636 (2014).
15. K.-H. Chao, K. Barton, S. Palmer, R. Lanfear, sangeranalyseR: Simple and Interactive Processing of Sanger Sequencing Data in R. *Genome Biol. Evol.* **13** (2021).
16. G. Marçais, C. Kingsford, A fast, lock-free approach for efficient parallel counting of occurrences of k-mers. *Bioinformatics* **27**, 764–770 (2011).
17. T. R. Ranallo-Benavidez, K. S. Jaron, M. C. Schatz, GenomeScope 2.0 and Smudgeplot for reference-free profiling of polyploid genomes. *Nat. Commun.* **11**, 1432 (2020).
18. H. Cheng, G. T. Concepcion, X. Feng, H. Zhang, H. Li, Haplotype-resolved de novo assembly using phased assembly graphs with hifiasm. *Nat. Methods* **18**, 170–175 (2021).
19. M. J. Roach, S. A. Schmidt, A. R. Borneman, Purge Haplotigs: allelic contig reassignment for third-gen diploid genome assemblies. *BMC Bioinformatics* **19**, 460 (2018).
20. E. V. Kriventseva, *et al.*, OrthoDB v10: sampling the diversity of animal, plant, fungal, protist, bacterial and viral genomes for evolutionary and functional annotations of orthologs. *Nucleic Acids Res.* **47**, D807–D811 (2019).
21. S. Ou, *et al.*, Benchmarking transposable element annotation methods for creation of a streamlined, comprehensive pipeline. *Genome Biol.* **20**, 275 (2019).
22. Z. Bao, *et al.*, Genome architecture and tetrasomic inheritance of autotetraploid potato. *Mol. Plant* **15**, 1211–1226 (2022).
23. D. Kim, J. M. Paggi, C. Park, C. Bennett, S. L. Salzberg, Graph-based genome alignment and genotyping with HISAT2 and HISAT-genotype. *Nat. Biotechnol.* **37**, 907–915 (2019).

24. K. J. Hoff, S. Lange, A. Lomsadze, M. Borodovsky, M. Stanke, BRAKER1: Unsupervised RNA-Seq-Based Genome Annotation with GeneMark-ET and AUGUSTUS. *Bioinformatics* [Preprint] (2016). Available at: http://dx.doi.org/10.1093/bioinformatics/btv661.
25. K. J. Hoff, A. Lomsadze, M. Borodovsky, M. Stanke, Whole-genome annotation with BRAKER. *Methods Mol. Biol.* **1962**, 65–95 (2019).
26. T. Brůna, K. J. Hoff, A. Lomsadze, M. Stanke, M. Borodovsky, BRAKER2: automatic eukaryotic genome annotation with GeneMark-EP+ and AUGUSTUS supported by a protein database. *NAR Genom Bioinform* **3**, lqaa108 (2021).
27. L. Gabriel, *et al.*, BRAKER3: Fully Automated Genome Annotation Using RNA-Seq and Protein Evidence with GeneMark-ETP, AUGUSTUS and TSEBRA. *bioRxiv* (2023). https://doi.org/10.1101/2023.06.10.544449.
28. M. Alonge, *et al.*, Automated assembly scaffolding using RagTag elevates a new tomato system for high-throughput genome editing. *Genome Biol.* **23**, 258 (2022).
29. M. Pertea, *et al.*, StringTie enables improved reconstruction of a transcriptome from RNA-seq reads. *Nat. Biotechnol.* **33**, 290–295 (2015).
30. T. Brůna, A. Lomsadze, M. Borodovsky, GeneMark-ETP significantly improves the accuracy of automatic annotation of large eukaryotic genomes. *Genome Res.* **34**, 757–768 (2024).
31. M. Stanke, M. Diekhans, R. Baertsch, D. Haussler, Using native and syntenically mapped cDNA alignments to improve de novo gene finding. *Bioinformatics* **24**, 637–644 (2008).
32. L. Gabriel, K. J. Hoff, T. Brůna, M. Borodovsky, M. Stanke, TSEBRA: transcript selector for BRAKER. *BMC Bioinformatics* **22**, 566 (2021).
33. F. Sevestre, M. Facon, F. Wattebled, N. Szydlowski, Facilitating gene editing in potato: a Single-Nucleotide Polymorphism (SNP) map of the Solanum tuberosum L. cv. Desiree genome. *Scientific Reports* [Preprint] (2020). Available at: http://dx.doi.org/10.1038/s41598-020-58985-6.
34. K. R. Amundson, *et al.*, Rare instances of haploid inducer DNA in potato dihaploids and ploidy-dependent genome instability. *Plant Cell* **33**, 2149–2163 (2021).
35. S. F. Altschul, W. Gish, W. Miller, E. W. Myers, D. J. Lipman, Basic local alignment search tool. *J. Mol. Biol.* **215**, 403–410 (1990).
36. A. R. Quinlan, I. M. Hall, BEDTools: a flexible suite of utilities for comparing genomic features. *Bioinformatics* **26**, 841–842 (2010).
37. R. van Berloo, R. C. B. Hutten, H. J. van Eck, R. G. F. Visser, An Online Potato Pedigree Database Resource. *Potato Res.* **50**, 45–57 (2007).
38. A. Cagan, *et al.*, Somatic mutation rates scale with lifespan across mammals. *Nature* **604**, 517–524 (2022).
39. M. Gerstung, E. Papaemmanuil, P. J. Campbell, Subclonal variant calling with multiple samples and prior knowledge. *Bioinformatics* **30**, 1198–1204 (2014).
40. R. E. Tarone, Testing the goodness of fit of the binomial distribution. *Biometrika* **66**, 585–590 (1979).
41. B. J. Ordoñez Aquino, “Novel genomic rearrangements derived from haploid induction and their potential use in plant breeding,” UC Davis, Davis, CA. (2023).
